# Machine learning prediction of intestinal α-glucosidase inhibitors using a diverse set of ligands: a drug repurposing effort with drugBank database screening

**DOI:** 10.1007/s40203-025-00384-8

**Published:** 2025-06-25

**Authors:** Adeshina I. Odugbemi, Clement Nyirenda, Alan Christoffels, Samuel A. Egieyeh

**Affiliations:** 1 South African Medical Research Council Bioinformatics Unit, South African National Bioinformatics Institute, , University of the Western Cape, Bellville, Cape Town, 7535 South Africa; 2 Department of Computer Science, University of the Western Cape, Cape Town, 7535 South Africa; 3 School of Pharmacy, University of the Western Cape, Bellville, Cape Town, 7535 South Africa; 4 National Institute for Theoretical and Computational Sciences (NITheCS), Stellenbosch, South Africa

**Keywords:** QSAR, Machine learning, Diabetes, α-glucosidase, Drug repurposing, Virtual screening

## Abstract

The global rise in diabetes mellitus (DM) poses a significant health challenge, necessitating effective therapeutic interventions. α-Glucosidase inhibitors play a crucial role in managing postprandial hyperglycemia and reducing the risk of complications in Type 2 DM. Quantitative Structure–Activity Relationship (QSAR) modelling is critical in computational drug discovery. However, many QSAR studies on α-glucosidase inhibitors often rely on limited compound series and statistical methods, restricting their applicability across wide chemical space. Integrating machine learning (ML) into QSAR offers a promising avenue for discovering novel therapeutic compounds by handling complex information from diverse compound sets. Our study aimed to develop robust predictive models for α-glucosidase inhibitors using a dataset of 1082 compounds with known activity against intestinal α-glucosidase (maltase-glucoamylase). After data preparation, we used 626 compounds to train ML models, generating different training data of three distinct molecular representations: 2D-descriptors, 3D-descriptors, and Extended-connectivity-fingerprint (ECFP4). These models, trained on random forest and support vector machine algorithms, underwent rigorous evaluation using established metrics. Subsequently, the best-performing model was used to screen the Drugbank database, identifying potential α-glucosidase inhibitor drugs. Drug repurposing, an expedited strategy for identifying new therapeutic uses for existing drugs, holds immense potential in this regard. Molecular docking and molecular dynamics simulations further corroborated our predictions. Our results indicate that 2D descriptors and ECFP4 molecular representations outperform 3D descriptors. Furthermore, drug candidates identified from DrugBank screening exhibited promising binding interactions with α-glucosidase, supporting our ML predictions and their potential for drug repurposing.

**Supplementary Information:**

The online version contains supplementary material available at 10.1007/s40203-025-00384-8.

## Introduction

Diabetes mellitus (DM), marked by hyperglycaemia, presents a significant global health challenge, with its prevalence steadily escalating worldwide. The number of global cases of diabetes is approximately 537 million and is projected to be over 637 million by 2030. DM manifests primarily as Type 1 DM, characterized by inadequate insulin production, and Type 2 DM, characterized by insulin resistance or insufficient insulin secretion, accounting for over 90% of cases (International Diabetes Federation 2021). The alarming rise in diabetes prevalence underscores the critical need for innovative therapeutic interventions.

One therapeutic strategy to manage Type 2 DM is targeting postprandial hyperglycaemia, the rise in blood glucose levels after meals, a significant contributor to vascular complications in Type 2 DM (Aryangat and Gerich 2010). Intestinal α-Glucosidase inhibitors are a class of drugs that achieve this by delaying carbohydrate digestion and absorption in the small intestine (Joshi et al. 2015). Targeting postprandial glucose levels with α-glucosidase inhibitors offers a promising avenue for managing glycaemic control and mitigating the risk of vascular complications. Another advantage of α-glucosidase inhibitors lies in their localized action at the brush border, potentially eliminating the need for systemic absorption to achieve therapeutic effects. While acarbose, an existing α-glucosidase inhibitor, is available, there's a need for novel inhibitors with improved efficacy and fewer side effects. Moreover, acarbose, a short oligosaccharide, has shown evidence of being degraded in the small intestine, which may impact its efficacy (Balaich et al. 2021; Tian et al. 2023).

Traditional drug discovery approaches have primarily relied on empirical screening, which can be laborious, time-consuming, and may yield compounds with limited efficacy or adverse effects. In recent years, the integration of machine learning techniques into drug discovery has emerged as an advancement in Quantitative Structure–Activity Relationship (QSAR) approaches (Keyvanpour & Shirzad 2021; Soares et al. 2022). This integration is a promising avenue for accelerating the identification and optimization of novel therapeutics. Previous QSAR studies on α-glucosidase inhibitors have often been confined to limited compound series, restricting the predictive reliability of models within a constrained chemical space (Ahmadi et al. 2022; Asadollahi-Baboli & Dehnavi 2018; Dahmani et al. 2021; Joshi et al. 2021; Kaur et al. 2018; Mora et al. 2018). To overcome this limitation, using structurally diverse ligands is imperative to develop models with broader applicability.

While QSAR studies abound in literature, including those on α-glucosidase inhibitors, the validation of these models often lacks utilization as tools for discovering new potential bioactive hits. Consequently, the literature is populated with statistically validated models, devoid of their primary objective: identifying and optimizing bioactive compounds. This occurrence highlights the need for researchers to demonstrate their models’ practical use as screening tools for identifying potential drug candidates (Abuhammad & Taha 2016).

Molecular representation is a critical aspect of developing QSAR models. This can be achieved through the generation of molecular descriptors, such as 2D descriptors, 3D descriptors, and molecular fingerprints (Kuz’min et al. 2021). While many studies utilize these representations separately or in combination, QSAR studies on α-glucosidase seldom undertake statistical comparisons between these molecular representations to establish their relative effectiveness.

This study addresses these gaps in the field. We aimed to develop machine learning QSAR models for α-glucosidase inhibitors from a more extensive and structurally diverse set of ligands. We built three datasets for model building: 2D and 3D molecular descriptors and Extended-Connectivity Fingerprints (ECFP). We then statistically compared the performance of models built across each representation. Finally, we employed the best-performing model to screen the Drugbank database for potential α-glucosidase inhibitor hits in a drug repurposing effort.

In this study, we identified drugs with repurposing potential against α-glucosidase. We also revealed that ECFP and 2D descriptor-based models achieved superior performance compared to 3D representations.

Subsequent sections in this research article outline the methods used to achieve our objective and findings. In Sect. “Methods”, we present details of our data preparation, model development and evaluation, virtual screening, and molecular modelling evaluation of identified hits. Our findings and discussion are presented in Sect. “Results and discussion”, and our conclusion is in Sect. “Conclusion”.

## Methods

### Dataset collection and preparation

In this study, we retrieved 1082 compounds from the PubChem database (Kim et al. 2024). These compounds have reported experimental bioassay data against intestinal alpha-glucosidase (UniProt ID: O43451). The PubChemPy (version 1.0.4) Python library was employed to extract the Simplified Molecular-Input Line Entry System (SMILES) data for each compound using their respective PubChem Compound Identifier (CID).

A meticulous data cleaning process was then implemented. First, compounds lacking an exact IC_50_ value (indicated by the absence of an "equals" activity qualifier) were excluded. Additionally, duplicate entries were removed to ensure a non-redundant dataset. Subsequently, the compounds underwent structural optimization using the LigPrep module from Schrödinger suite 2021–4 release (Schrödinger, LLC, New York, NY, 2021). Following these comprehensive data preparation steps, a total of 626 compounds were deemed suitable for further analysis and machine learning model development.

As a subsequent step, the IC_50_ values were transformed into pIC_50_ values by taking the negative logarithm (base 10) of the original values. This transformation addressed the vast range of IC_50_ values observed within the dataset (0.00579 μM–1000 μM). The pIC_50_ scale provided a more manageable and machine learning-friendly distribution (3.00–8.24). Analysis of the pIC_50_ trend revealed a distinct threshold (pIC_50_ = 5.69) that served to categorize the compounds into active and inactive classes. Compounds with pIC_50_ values greater than or equal to the threshold were classified as active, while those below were classified as inactive.

### Molecular featurization

Molecular featurization is the process of converting a compound’s molecular structure into a fixed-length feature vector suitable for machine learning algorithms. These vectors serve as the independent variables for subsequent modelling. In this study, three distinct datasets were generated from the compounds’ dataset, each containing fixed-length feature vectors representing the compounds. These datasets captured different numerical representations of the molecular structure: (i) 2D descriptors, (ii) 3D descriptors, and (iii) Extended-connectivity fingerprint.

The workflow involved generating a Standard Data Format (SDF) file for each compound using DataWarrior software (Sander et al. 2015) (https://openmolecules.org/datawarrior). These SDF files were then used as input for the PaDEL application (a Python wrapper for PaDEL-Descriptor software, version 0.1.16) to compute the 2D and 3D descriptors (Yap 2011). Extended-connectivity fingerprint of 4 bonds diameter (ECFP4) was generated using RDKit, an open-source cheminformatics toolkit (Landrum et al. 2020).

Prior to training machine learning models, t-distributed stochastic neighbour embedding (t-SNE) plots were generated for each featurized dataset. This visualization technique aided in exploring the high-dimensional data and identifying potential relationships between the compounds based on their feature vectors (Soni et al. 2020). Following this initial exploration, each featurized dataset was then used to train machine learning models.

### Data splitting

Data splitting is a crucial step in machine learning, where the original dataset is partitioned into training and test sets. In this study, each featurized dataset (2D descriptors, 3D descriptors, and ECFP fingerprints) was meticulously divided into training and test sets using a ratio of 80% and 20%, respectively. This split aimed to ensure a representative sample for model training while reserving a portion for unbiased evaluation.

To maintain the integrity of the data, RDKit was employed during the splitting process. This ensured that the training and test sets preserved the original dataset’s structural diversity and the distribution of activity labels. To verify the uniformity of similarity between the sets, the average Tanimoto similarity coefficient was calculated for both training and test sets. Additionally, DataWarrior software was utilized to visually assess the similarity landscapes of the training and test sets.

### Feature selection

Forward feature selection was initiated using a sequential feature selector on both the 2D and 3D datasets. This approach aimed to identify a relevant subset of features from the larger pool generated. The primary goals were to reduce model complexity and potentially mitigate overfitting. The selection process consisted of three steps. First, descriptors with constant or low variance (threshold = 0.01) were removed. Second, redundant descriptors were eliminated by removing one member from each pair exhibiting a correlation coefficient greater than 0.85. Finally, these pre-processed features were fed into the sequential feature selection process. The mlxtend library’s sequential feature selector was utilized to iteratively evaluate feature subsets using tenfold cross-validation (Raschka 2018). This step aimed to identify the minimum feature subset that achieved the highest accuracy. Due to computational demands, the final step was executed on high-performance computing resources. The specific parameters employed by the sequential feature selector are detailed in Table 1.

**Table 1:** Parameter settings for 2D and 3D feature selection

Parameter	Option	Description
k_features	Best	Number of features to select, where k_features < the full feature set. Where "best" is provided, the feature selector will return the feature subset with the best cross-validation performance
Forward	True	Forward feature selection approach is used
Estimator	Random forest classifier	A scikit-learn classifier which trains multiple decision tree classifiers on different subsets of the dataset, employing averaging to enhance predictive accuracy
Scoring	Balanced accuracy	The scoring metric from the estimator. It is the average of recall obtained on each class, dealing with imbalanced datasets
Cross Validation	tenfold	The dataset is divided into 10 equal parts, and the model is trained and evaluated 10 times, each time using a different part as the validation set and the remaining data as the training set

### Model development and optimization

In this study, we use Random Forest (RF) and Support Vector Machine (SVM) as our primary machine learning algorithms due to their proven reliability and interpretability in cheminformatics. RF is effective for handling high-dimensional descriptor data and is robust to overfitting, while SVM is known for its performance in classification tasks with moderate sample sizes (Odugbemi et al. 2024). Both algorithms have a strong track record in QSAR modelling and bioactivity prediction, and are conveniently available in scikit-learn, a widely used open-source Python library (Pedregosa et al. 2011).

#### Hyperparameter tuning

Machine learning algorithms have configuration settings known as hyperparameters that influence how the model learns from data. In this study, we did not rely on the default hyperparameter settings of the selected algorithms to build ML models. Instead, we performed hyperparameter tuning to optimize model performance for our dataset. This decision acknowledges the importance of hyperparameter tuning, which optimizes model performance for a specific dataset. To achieve this, a grid search approach was implemented using the GridSearchCV module from scikit-learn. This method systematically evaluates a predefined grid of hyperparameter values for each classifier. The optimal hyperparameter combination is identified through cross-validation, ensuring the model's generalizability to unseen data. The evaluation metric used to select the best performing set of hyperparameters was the minimization of the log-loss function, which incorporates the predicted probabilities for each class, rather than the sole consideration of the predicted class labels. This metric evaluates whether predictions are correct, and how confident the model is in its predictions, making it more informative than simple accuracy for classification tasks.

#### Model building and validation

Following hyperparameter tuning, the machine learning algorithms, Random Forest (RF) and Support Vector Machine (SVM), were used to develop our models. These algorithms have a well-established reputation for effectiveness in cheminformatics and machine learning-driven drug discovery research, as demonstrated in numerous publications (Kurniawan et al. 2022; Shi 2021; Trinh et al. 2022; Zhou et al. 2021). Each model was trained using the respective training set, and its performance was rigorously evaluated using a combination of internal and external validation techniques.

Internally, a tenfold cross-validation was implemented on the training set for each model. This method iteratively splits the training data into ten folds, trains the model on nine folds, and validates its performance on the remaining fold. This process is repeated ten times, providing a robust assessment of the model's consistency and avoiding overfitting. While tenfold cross-validation was employed on the training set for internal validation, a strictly separate test set was reserved for external validation. During each cross-validation iteration, performance metrics were calculated on both the training and validation folds. However, the assessment of model generalizability was based on its performance on the unseen test set, which remained untouched throughout the cross-validation process.

For both the training and test sets, the performance of each model was evaluated using a confusion matrix. This is a tabular representation that compares the model’s predicted class labels to the actual labels (Table 2). From the confusion matrix, various metrics were calculated to provide a comprehensive understanding of model performance, including accuracy, balanced accuracy, F1 score, and Matthews Correlation Coefficient (MCC). These metrics consider different aspects of a model's performance, such as its ability to correctly classify both positive and negative cases, and its robustness to imbalanced datasets.

**Table 2:** Confusion matrix

	Predicted negative	Predicted positive
Predicted negative	True negative (TN)	False positive (FP)
Predicted positive	False negative (FN)	True positive (TP)

Additionally, the Receiver Operating Characteristic curve and its Area Under the Curve (ROC-AUC) were generated for each model. The ROC-AUC provides a metric that summarizes the model's ability to discriminate between positive and negative classes.

*Accuracy*. A common metric which is calculated by dividing the number of correct predictions (both positive and negative) by the total number of instances.

1 $$\text{Accuracy}=\frac{\text{TP}+\text{TN}}{\text{TP}+\text{TN}+\text{FP}+\text{FN}}$$

*Balance accuracy*. It is a variation of the accuracy metric that takes into account imbalanced datasets, as is in our case. It addresses the shortcomings of traditional accuracy, which can be misleading in imbalanced datasets where the model could simply classify everything as the majority class to achieve high accuracy.

2 $$\text{Balanced Accuracy}=\frac{1}{2}\left(\frac{\text{TP}}{\text{TP}+\text{FN}}+\frac{\text{TN}}{\text{TN}+\text{FP}}\right)$$

*F1 score*. It is the harmonic mean of precision and recall, and it ranges from the worst score of 0 to the perfect score of 1. It is also useful in situations where there is an imbalance between the number of positive and negative instances in the dataset.

3 $$\text{F}1\_\text{score}=\frac{2\times \text{TP}}{2\times \text{TP}+\text{FP}+\text{FN}}$$

*Matthews Correlation Coefficient*. It considers all four confusion matrix elements, which makes it more robust to class imbalance compared to other metrics. MCC ranges from -1 (perfect disagreement) to 1 (perfect agreement), and 0 indicates random guessing.

4 $$\text{MCC}=\frac{\text{TP}\times \text{TN}-\text{FP}\times \text{FN}}{\sqrt{\left(TP+FP\right)\left(TP+FN\right)\left(TN+FP\right)\left(TN+FN\right)}}$$

*Log-loss*. Logarithmic Loss, commonly known as log-loss or cross-entropy loss, is a metric used to evaluate the performance of a classification model, particularly in the context of probabilistic predictions. It measures the difference between the predicted probabilities and the actual labels, penalizing models more severely for confidently wrong predictions. Log-loss incentivizes models to accurately distinguish active from inactive molecules, particularly in virtual screening tasks. Lower log-loss indicates better model performance, and a model with perfect skill has a log-loss score 0.

5 $$\text{Log Loss}=-\frac{1}{N}{\sum }_{i=1}^{N}[{y}_{i}\text{log}\left({p}_{i}\right)+\left(1-{y}_{i}\right)\text{log}(1-{p}_{i})]$$

where:

${\varvec{N}}$ = the number of samples in the dataset.

${{\varvec{y}}}_{{\varvec{i}}}$ = the actual binary label for the *i-th* sample (0 or 1).

${{\varvec{p}}}_{{\varvec{i}}}$ = the predicted probability that the i-th sample belongs to class 1.

Analysis of variance (ANOVA) on the RF tuned models’ evaluation metrics performance on the validation set of each iteration in the tenfold cross-validation process was performed. The Tukey’s post hoc test was used for comparing the means of all dataset pairs.

#### Learning curves

Scikit-learn's learning curve module was utilized to assess the learning behaviour of each model. Learning curves visualize the model's performance as the training data size increases. This analysis helps identify potential overfitting and the point of diminishing returns, informing decisions about the adequacy of training data.

#### Y-randomization test

To assess model robustness against chance correlations, a Y-randomization test was performed. In this test, the activity labels in the training data were shuffled, disrupting the relationship between the features and the target variable. The resulting data with permuted labels was then used to train a new model, and its performance was compared to the original model trained on the unpermuted data.

#### Applicability domain

To ensure the reliability of predictions, the applicability domain (AD) of a chosen top-performing model was established. The AD defines the chemical space where the model's predictions can be considered trustworthy. Euclidean distance was employed to assess the similarity of new data points to the training data used to develop the model. The AD threshold is calculated as follows (Zhang et al. 2006):

6 $$\text{AD}=d+\left(z\times \sigma \right)$$

where,

$$\mathbf{A}\mathbf{D}$$ = Applicability Domain.

${\varvec{d}}$ = Euclidean distance of a compound in the descriptor space.

${\varvec{z}}$ = Z-score, a measure of how many standard deviations a data point is from the mean.

${\varvec{\sigma}}$ = Standard deviation of the descriptor values in the training set.

The Python scripts for training the models are available on https://github.com/shinoxide/ML_QSAR_model.

### Virtual screening

The top-performing machine learning model was applied to virtual screening aimed at identifying potential drug candidates. The Drugbank database (version 5.1.10) (Wishart et al. 2018), accessed via the Probes and Drugs platform (Skuta et al. 2017), served as the screening library. Drugs from Drugbank were evaluated using the established AD of the model. Only compounds with Euclidean distances falling within the AD threshold were retained for virtual screening.

### Molecular modelling of identified hits

The identified hits were subjected to molecular docking and molecular dynamics simulation with the structural model of intestinal α-glucosidase (2QMJ) (Sim et al. 2008). The protein structure was prepared using the Protein Preparation Wizard in the Schrodinger Suite 2021–4 release (Schrödinger, LLC, New York, NY, 2021) and the hits prepared using the LigPrep module within the same suite. Energy minimisation was performed using the Optimized Potentials for Liquid Simulations (OPLS4) force field (Lu et al. 2021). An extra precision (XP) docking approach was used to dock the prepared ligands into the prepared target protein and the docking computations also used the OPLS4 force field. The Prime-MMGBSA panel of the Prime module in the Schrodinger Suite was used to calculate ligand binding free energies using molecular mechanics with generalized Born and surface area solvation (MM-GBSA) approach. The variable-dielectric generalized Born model (VSGB) was used as the implicit solvation model.

The molecular dynamics simulations were performed with the Desmond package in the Schrodinger Suite 2021–4 release as described in a previous study (Ojo et al. 2023). The system was first set up with the System Builder module in the Desmond package. The docked complexes were each placed in an orthorhombic box with a buffered distance of 10 Å beyond the complexes. The complexes were solvated with a four-site water model, TIP4P. The system was neutralised by adding sodium and chloride counter ions and was then set at 0.15 M NaCl to mimic the physiological state. The system was equilibrated with NPT ensemble class at a temperature of 310 K and a pressure of 1.01325 bar. The simulation was performed for 150 ns, with trajectories recorded every 100 ps, for a total output of 1500 frames. The simulation’s time step was set to 2.0 fs. After simulations, the free binding energies were estimated on the trajectories using the MM-GBSA approach.

## Results and discussion

### Datasets, labeling and molecular featurization

**Fig. 1:**
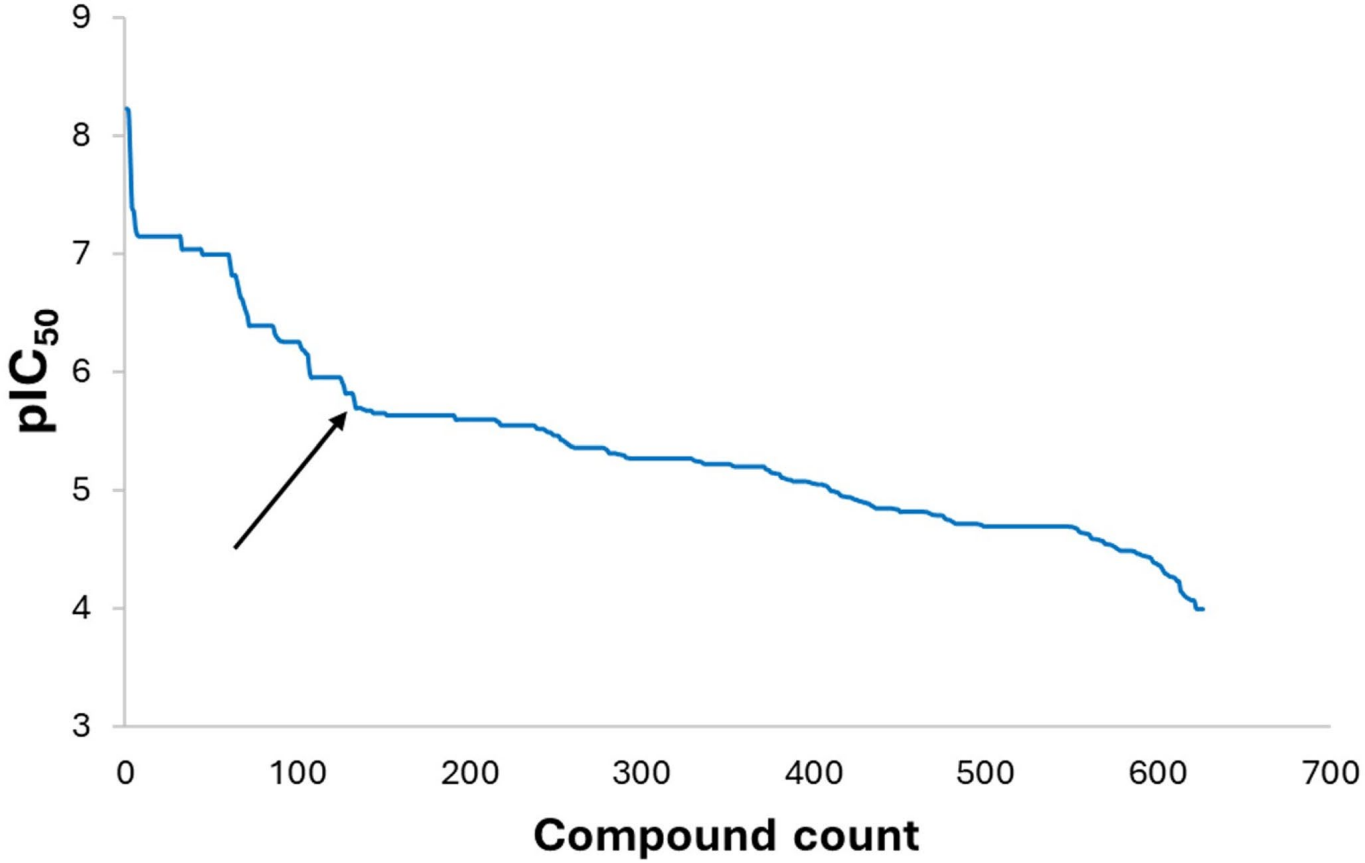
Plot of pIC_50_ trend in the dataset, showing threshold of pIC50 5.69, 2.04 µM (black arrow) used to establish categorization of the dataset into active and inactive compounds

An initial dataset of 1082 compounds with reported activity against intestinal alpha-glucosidase was retrieved from PubChem. Following a filtering process detailed in Sect. “Methods”, a total of 626 compounds were deemed suitable for further analysis and machine learning model development (prepared dataset provided in supplementary file 2). To enable binary classification, IC₅₀ values were first transformed to pIC₅₀. Analysis of the pIC₅₀ distribution (Fig. 1) revealed a noticeable inflection point nearer to the centre that is marked by a sharp transition from a steeper to a flatter slope at pIC₅₀ 5.69. This point was selected as the threshold to categorize compounds into active (pIC₅₀ ≥ 5.69, n = 138; 22%) and inactive (pIC₅₀ < 5.69, n = 488; 78%). This binary classification scheme aimed to facilitate a clearer learning process for the machine learning models.

Three distinct datasets were generated from the preprocessed dataset of 626 compounds to represent the molecular structural features for machine learning models. PaDEL software was employed to generate a set of 1444 descriptors capturing various aspects of the two-dimensional (2D) structure of each compound. Similarly, PaDEL was used to generate a set of 431 descriptors representing the 3D structural features of the compounds. A few compounds produced null values during 3D descriptor calculation in PaDEL, likely due to geometry or structure-related issues. To avoid descriptor inconsistencies across the dataset, we excluded these compounds from the 3D descriptor dataset prior to model training. This resulted in a reduced 3D descriptor set of 605 compounds. Finally, RDKit was utilized to compute Extended-connectivity fingerprints (ECFP4) for each compound. ECFP4 fingerprints represent each compound as a 2048-bit vector, where each bit signifies the presence or absence of a specific circular substructure within the compound.

**Fig. 2:**
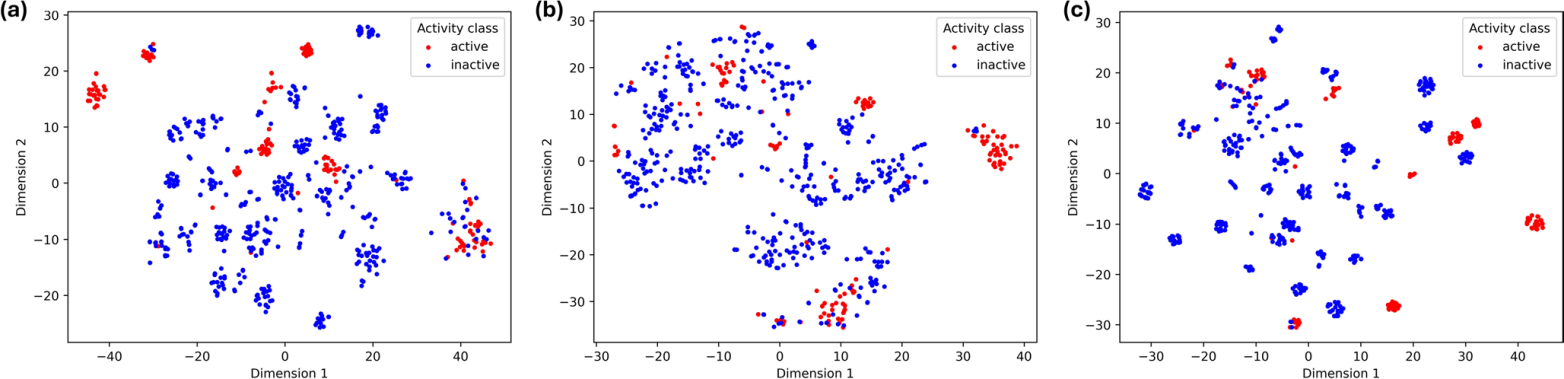
t-SNE plots for **a** 2D descriptor set **b** 3D descriptor set and **c** ECFP set. The red and blue dots represent active and inactive activity label respectively. The 2D descriptor and ECFP4 representations revealed more distinct clusters for the active chemicals than the 3D representation.

Two-dimensional t-SNE was used to visualize the relationships between the high-dimensional molecular representations in each dataset. This visualization is crucial for understanding the inherent structure of the data, identifying clustering patterns, and gaining insights into the relationships between compounds based on their feature representations (Balamurali 2020; Soni et al. 2020). The t-SNE as opposed to a popular technique, principal component analysis, excels at capturing non-linear relationships between features (Anowar et al. 2021), leading to a more accurate visual representation of QSAR data (Fig. 2).

### Data splitting and assessment of structural diversity across training and test sets

In machine learning, splitting a dataset into training and test sets (often in an 80/20 ratio) is a critical step for model building. The training set is used to train the model, allowing it to learn patterns from the data and adjust its internal parameters. The unseen test set then evaluates the model's ability to generalize and perform well on data it hasn't encountered during training.

This splitting process, performed using RDKit, emphasizes the importance of ensuring both training and test sets fairly represent the original dataset. To this end, we considered both the chemical structures and activity in the splitting, checking the average Tanimoto similarity across the resulting train and test sets. Tanimoto similarity is a metric that measures the chemical similarity between molecules. By ensuring similar average similarity in both sets, we confirm that the training and test sets are not biased towards specific types of molecules, which could lead to misleading performance evaluations. (Sheridan et al. 2004; Simm et al. 2021). The average similarity across training and test sets are observed to be similar as shown in Table 3.

**Table 3:** Average Tanimoto similarity across datasets before and after splitting

	2D	3D	ECFP4
Whole data	0.128	0.126	0.127
Training data	0.126	0.127	0.127
Test data	0.130	0.124	0.127

**Fig. 3:**
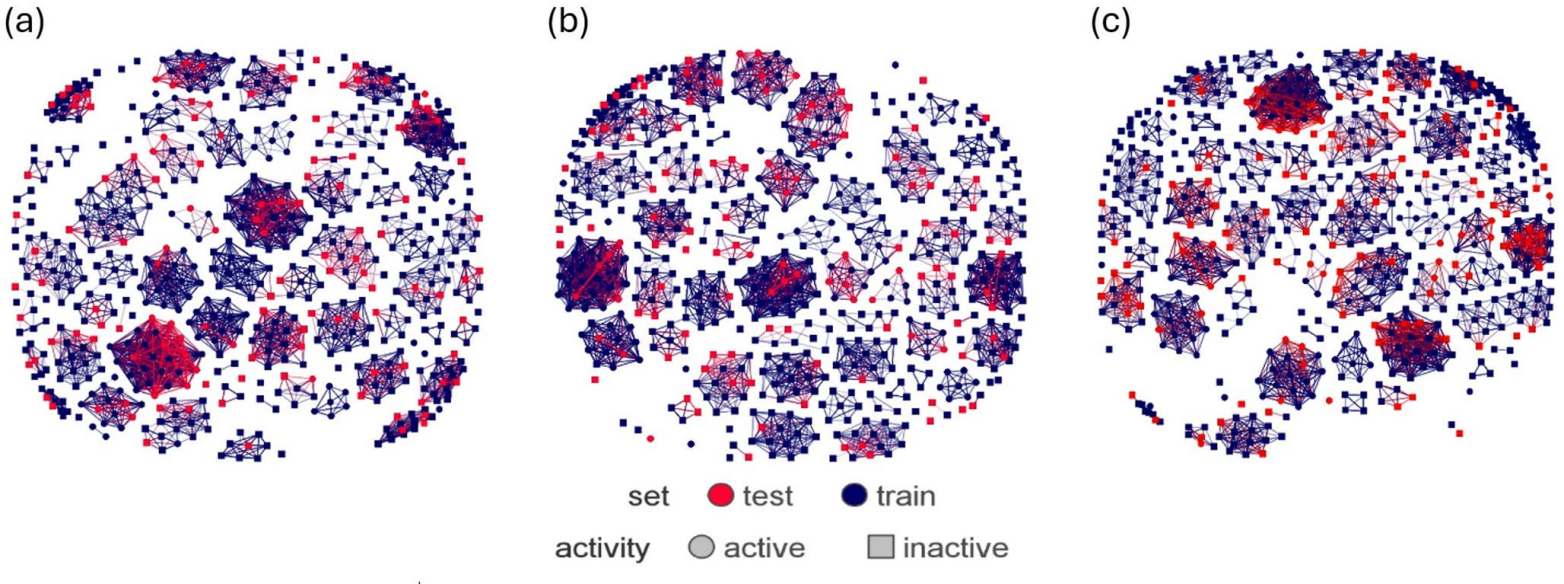
Structural similarity distributions across **a** 2D descriptor **b** 3D descriptor and **c** ECFP4 datasets, showing relative uniform distribution of training and test compounds across clusters.

To visually assess structural distribution of the datasets, DataWarrior was used to create a similarity chart of the compounds in the datasets. The training and test sets were well represented (dark blue and red points respectively), widely spread across structural similarity clusters, as shown in Fig. 3. The figure further shows that the compounds are clustered by activity, with circular points representing actives and the square points representing inactives. This suggests that the activity labels are finely divided along structural groups making the dataset machine learning friendly. However, a few activity cliffs are observed, where a compound or two within a cluster have different activity from the rest of the compounds within the same cluster.

### Feature selection

Forward feature selection was performed on the 2D and 3D dataset. This was essentially used to select a subset of relevant features from the larger pool of features generated, primarily aiming to reduce model complexity and potentially avoid overfitting (Redkar et al. 2020). The list of the selected features is presented in Table 4. Feature selection was not performed on the ECFP4 dataset. ECFP4 fingerprints represent molecules by encoding the local environment surrounding each atom. Since ECFP4 focuses on local environments, the features are inherently less redundant. Each fingerprint element describes a specific atomic neighbourhood, and removing such features could lead to a loss of information about the molecule's structure.

**Table 4:** Selected features from the 2D and 3D descriptor dataset using forward feature selection

	Selected features
2D	*nN, nF, GATS5c, GATS6s, SpMax1_Bhv, C3SP2, nHBint4, nHdCH2, nssCH2, naasN, nssS, StCH, minHBint10*
3D	*TDB4u, TDB5u, TDB1e, TDB2s, TDB5s, TDB7s, PNSA-1, PNSA-3, RPCS, THSA, TPSA, RDF30u, RDF20m, RDF40m, L2u, E3u, Dm*

### Development and evaluation of supervised learning classification models

Initial models’ hyperparameters were optimized in a fivefold cross-validation experiment on a subset of the training set. This optimization was necessary to select the best combination of hyperparameters for the ML models using the Scikit-learn GridSearchCV algorithm. Each of the dataset was fitted on RF and SVM classifiers with the best parameters from the GridSearchCV. The hyperparameter settings and the resulting optimum parameters for each dataset are presented in Table 5.

**Table 5:** Hyperparameter tuning of RF and SVM models

	Hyperparameters tuned	Optimum hyperparameters
RF	{'class_weight': [None,'balanced','balanced_subsample'],'max_depth': [None, 5, 10, 15, 20],'min_samples_leaf': [2, 3, 5, 7, 9],'min_samples_split': [3, 4, 6, 8, 10],'n_estimators': [30, 50, 100, 200]}	Trained on 2D descriptors: {'class_weight': None,'max_depth': None,'min_samples_leaf': 2,'min_samples_split': 3,'n_estimators': 200}
		Trained on 3D descriptors: {'class_weight': None,'max_depth': 15,'min_samples_leaf': 2,'min_samples_split': 3,'n_estimators': 30}
		Trained on ECFP4 fingerprint: {'class_weight': None,'max_depth': None,'min_samples_leaf': 2,'min_samples_split': 3,'n_estimators': 100}
SVM	{'C': [0.01, 0.1, 1, 10, 30],'kernel': ['linear','rbf','poly'],'gamma': ['scale','auto', 0.001, 0.01, 0.1],'degree': [1, 2, 3, 4],'coef0': [0.0, 0.1, 0.5],'shrinking': [True, False]}	Trained on 2D descriptors: {'C': 10,'coef0': 0.0,'degree': 1,'gamma': 0.1,'kernel':'rbf','shrinking': True}
		Trained on 3D descriptors: {'C': 10,'coef0': 0.0,'degree': 1,'gamma': 0.1,'kernel':'rbf','shrinking': True}
		Trained on ECFP4 fingerprint: {'C': 1,'coef0': 0.5,'degree': 3,'gamma': 0.01,'kernel':'poly','shrinking': False}

To assess the predictive performance of the models, we used the two standard approaches of model validation which are internal validation and external validation. A tenfold cross-validation approach was used for the internal validation. The reported metric scores presented are the mean ± std values of the collected scores from 10 iterations on the training/validation runs. The final model scores were obtained by testing the predictive power of the models on unseen data (test set), which was not included in the CV iterations. The metric scores used to assess models’ performance were generated from the results of the confusion matrix, which is a 2 × 2 matrix used to evaluate the performance of a classification model. It provides a summary of the predictions made by the model compared to the actual labels. The resultant confusion matrices are presented in Figure S1 and S2 (supplementary file 1). From these matrices, various performance metrics can be derived, including accuracy, among others (Eq. 1–4). While accuracy remains a popular choice, it's not always the most reliable as it may be misleading in imbalanced datasets as in our case. Therefore, in addition to accuracy, we have also reported metrics that are more robust to dataset imbalance, which includes balanced accuracy, F1-scores and Mathew’s correlation coefficient (MCC) scores. Balanced accuracy ensures each class contributes equally to the evaluation, addressing accuracy’s limitation. F1-score, on the other hand, captures the harmonic mean of precision and recall, providing a balanced view of model performance. Importantly, the MCC has been reported to possess superior assessment power for imbalanced datasets such as ours. The performance scores of all models, as assessed by accuracy and balanced accuracy, are presented in Table 6, and as assessed by the F1-score and MCC, are presented in Table 7. Here, we also compared the performance of models that have their hyperparameters tuned with the ones left in default mode. It was observed that the tuned models reduced overfitting tendencies that were recorded in untuned models. In essence, the gap between metric scores on training, validation and test sets were reduced, showing that overfitting was minimized.

**Table 6:** Cross-validation accuracy and balanced accuracy and model performance on unseen test set

		Accuracy			Balanced accuracy		
		Train	Validation	Test	Train	Validation	Test
2D	RF	1.00 ± 0.00	0.97 ± 0.02	0.95	1.00 ± 0.00	0.95 ± 0.03	0.89
	RF Tuned	0.98 ± 0.00	0.96 ± 0.03	0.96	0.96 ± 0.00	0.94 ± 0.05	0.92
	SVM	0.94 ± 0.01	0.92 ± 0.03	0.92	0.91 ± 0.02	0.86 ± 0.07	0.89
	SVM tuned	0.98 ± 0.00	0.95 ± 0.04	0.94	0.97 ± 0.01	0.92 ± 0.05	0.90
3D	RF	1.00 ± 0.00	0.93 ± 0.05	0.91	1.00 ± 0.00	0.89 ± 0.07	0.87
	RF tuned	0.99 ± 0.00	0.92 ± 0.04	0.91	0.97 ± 0.01	0.84 ± 0.07	0.86
	SVM	0.92 ± 0.00	0.89 ± 0.05	0.92	0.83 ± 0.01	0.78 ± 0.10	0.82
	SVM tuned	1.00 ± 0.00	0.92 ± 0.04	0.92	0.99 ± 0.00	0.88 ± 0.07	0.85
ECFP4	RF	1.00 ± 0.00	0.96 ± 0.02	0.95	1.00 ± 0.00	0.93 ± 0.05	0.91
	RF tuned	0.97 ± 0.00	0.96 ± 0.02	0.95	0.96 ± 0.01	0.93 ± 0.05	0.91
	SVM	0.97 ± 0.00	0.96 ± 0.02	0.95	0.96 ± 0.01	0.93 ± 0.04	0.91
	SVM tuned	0.97 ± 0.00	0.96 ± 0.02	0.94	0.95 ± 0.00	0.94 ± 0.05	0.87

**Table 7:** Cross-validation F1-score and MCC, and model performance on unseen test set

		F1-score			MCC		
		Train	Validation	Test	Train	Validation	Test
2D	RF	1.00 ± 0.00	0.94 ± 0.05	0.88	1.00 ± 0.00	0.93 ± 0.06	0.86
	RF Tuned	0.96 ± 0.00	0.92 ± 0.07	0.91	0.95 ± 0.01	0.90 ± 0.08	0.88
	SVM	0.86 ± 0.02	0.80 ± 0.09	0.82	0.83 ± 0.02	0.76 ± 0.10	0.77
	SVM Tuned	0.95 ± 0.01	0.88 ± 0.08	0.87	0.93 ± 0.01	0.85 ± 0.10	0.84
3D	RF	1.00 ± 0.00	0.83 ± 0.11	0.79	1.00 ± 0.00	0.80 ± 0.13	0.74
	RF Tuned	0.96 ± 0.01	0.78 ± 0.11	0.78	0.96 ± 0.01	0.74 ± 0.12	0.73
	SVM	0.78 ± 0.01	0.68 ± 0.17	0.77	0.74 ± 0.01	0.64 ± 0.19	0.74
	SVM Tuned	0.99 ± 0.01	0.81 ± 0.11	0.79	0.99 ± 0.01	0.76 ± 0.13	0.75
ECFP4	RF	1.00 ± 0.00	0.90 ± 0.06	0.88	1.00 ± 0.00	0.87 ± 0.07	0.86
	RF Tuned	0.94 ± 0.01	0.90 ± 0.07	0.88	0.92 ± 0.01	0.88 ± 0.07	0.86
	SVM	0.94 ± 0.01	0.91 ± 0.06	0.88	0.92 ± 0.01	0.88 ± 0.07	0.86
	SVM Tuned	0.93 ± 0.01	0.91 ± 0.06	0.84	0.91 ± 0.01	0.90 ± 0.07	0.81

Models that are trained on the 2D molecular descriptors or on the ECFP4 performed substantially better than those trained on the 3D molecular descriptors. For example, the MCC validation and test scores of the RF tuned model trained on the 2D molecular descriptors is given by 0.90 ± 0.08 and 0.88 respectively, and RF tuned model trained on the ECFP4 given by 0.88 ± 0.07 and 0.86. These scores are superior to the MCC scores of RF tuned model trained on 3D descriptors given by 0.74 ± 0.12 and 0.73. A similar trend is observed in the MCC for SVM tuned models across the three datasets, with 2D and ECFP4 models superior to 3D models.

A step further in the model’s performance assessment is evaluation of the Receiver Operating Characteristic—Area Under the Curve (ROC-AUC). The ROC-AUC holds significant importance in assessing the efficacy of machine learning models, particularly in binary classification tasks. It provides a holistic measure of a model's capacity to differentiate between classes, with higher ROC-AUC scores indicating superior discrimination performance. Moreover, the Mean ± Std of ROC-AUC across cross-validation folds offers insights into the model's stability and consistency across different data subsets or training iterations. This assessment helps in gauging the robustness of the model's performance and enhances confidence in its reliability under various conditions, thereby aiding in informed decision-making during model selection and deployment.

**Fig. 4:**
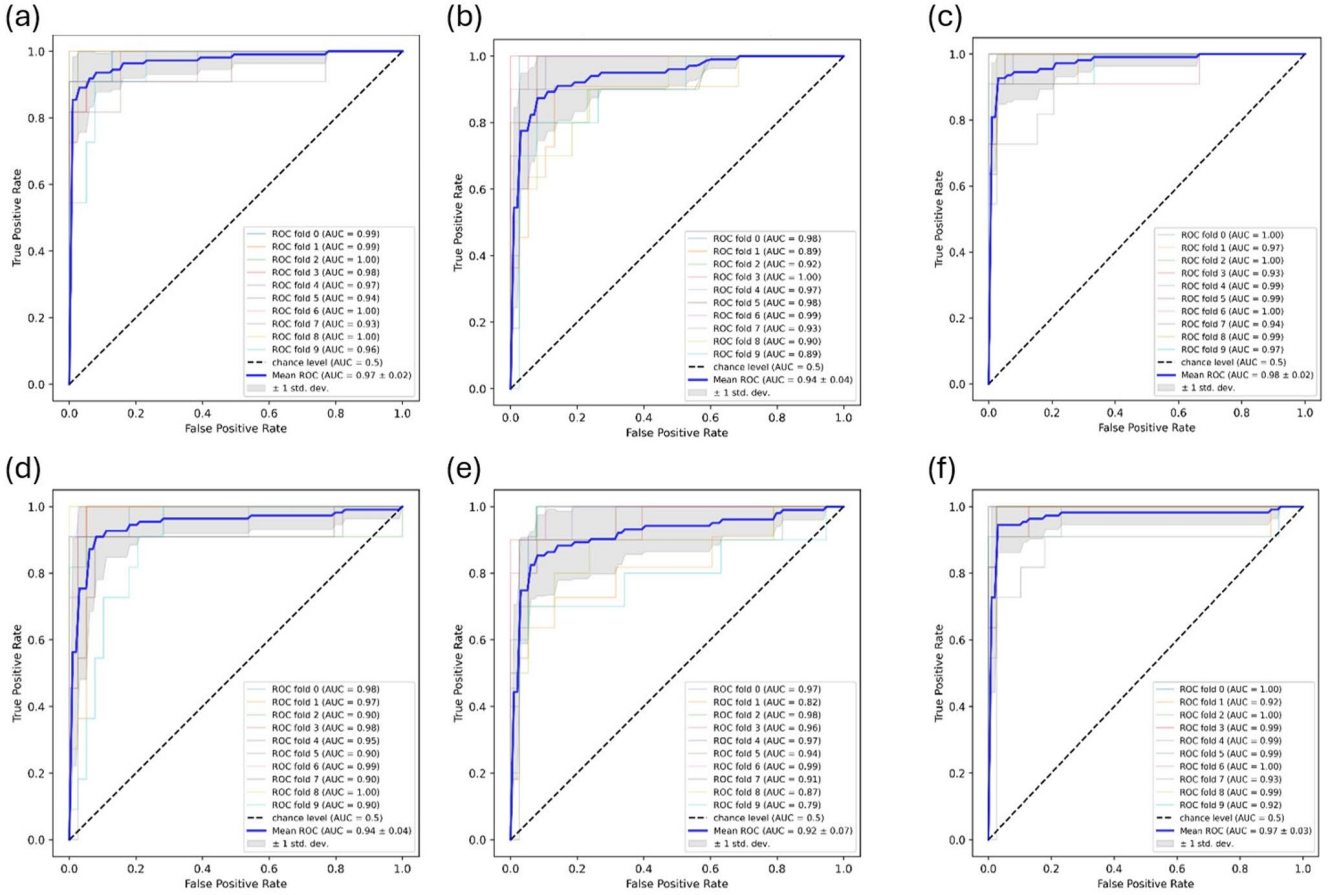
The ROC_AUC for predictions on validation set across each iteration of the tenfold CV for the tuned **a** RF 2D descriptors **b** RF 3D descriptors **c** RF ECFP4 **d** SVM 2D descriptors **e** SVM 3D descriptors and **c** ECFP4 fingerprint datasets

The ROC_AUC for predictions on validation set was plotted for each iteration of the tenfold CV and the result for the tunned models are presented in Fig. 4. The tuned RF model trained on ECFP4 has the highest average AUC with the least standard deviation across the CV iterations (0.98 ± 0.02), and this is followed by the tuned RF model trained on 2D descriptors (0.97 ± 0.02).

**Fig. 5:**
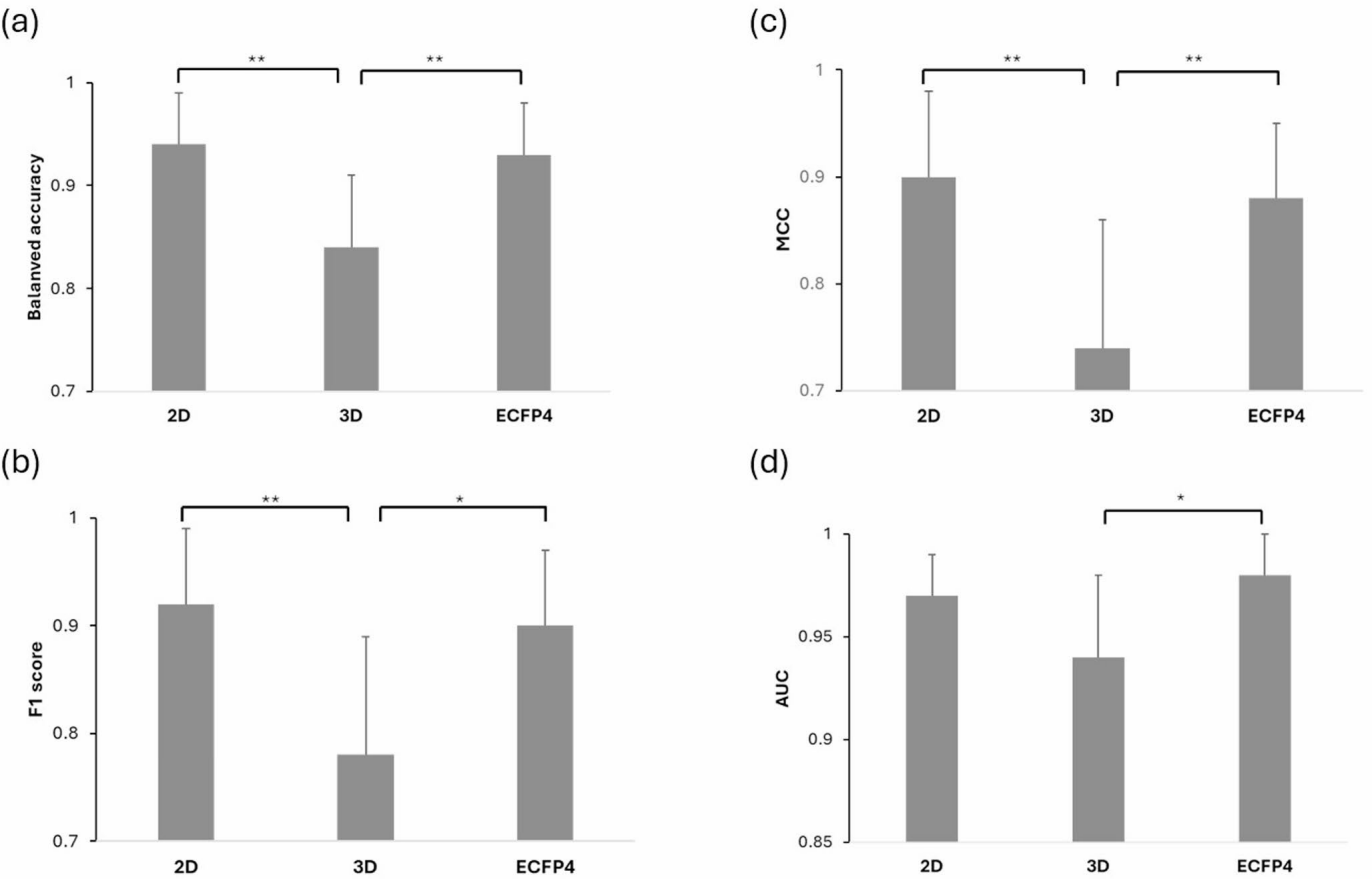
Assessment of the difference in evaluation metrics across the 2D descriptors, 3D descriptors and ECFP4 dataset. **a** Balanced accuracy **b** F1 score **c** MCC **d** AUC. *post test statistical difference (*p < 0.05, **p < 0.01)

Statistical analysis on the RF tuned models’ evaluation metrics performance on the validation set of each iteration in the tenfold cross-validation process is presented in Fig. 5. On performing ANOVA with Tukey’s post-test, ECFP4 and 2D descriptors have significantly higher metric performance than the 3D descriptors.

We may have expected that models trained on 3D descriptors will outperform those trained on 2D descriptors and the ECFP4 fingerprint, also based on 2D structural information. This expectation is because 3D descriptors have richer information, encompassing the spatial arrangement of atoms in a molecule and, hence, a more realistic representation. However, some factors, such as data sparsity and simplicity of structural representation, may explain why our models trained on 2D molecular descriptors and ECFP4 might perform better than those trained on 3D molecular descriptors from the same set of ligands.

ECFP4 result in more sparse data representations compared to 3D descriptors (Axen et al. 2017; Wigh et al. 2022). A sparse representation means that only a small fraction of the possible features is active or has non-zero values for a given molecule. ECFP fingerprints represent each molecule as a fixed-length binary vector. Each bit in this vector indicates the presence or absence of a specific substructure or structural pattern within the molecule. The sparsity in ECFP arises because each molecule in a dataset typically contains only a small subset of all possible structural features. Sparsity may be beneficial for model performance, especially when dealing with high-dimensional data such as ECFP fingerprints (Kensert et al. 2018; Probst & Reymond 2018). With fewer features in the high dimension influencing the model, it may be less prone to overfitting on training data, as we have seen in our study, making the model focus on these important features and perform better on unseen data.

Furthermore, 2D descriptors and ECFP fingerprints offer a simpler and more interpretable representation of chemical features compared to 3D descriptors. This advantage in simplicity and interpretability may translates to greater ease of understanding and better model performance (Asahara & Miyao 2022). 3D descriptors might introduce relatively unnecessary complexity by incorporating details about the molecule’s spatial arrangement that may not directly translate to activity. Moreover, a single conformation of the 3D molecular structure of each compound is used to develop machine-learning models based on 3D descriptors, and this may have implications for target-specific predictions (Vitorović-Todorović et al. 2012; Wilkes et al. 2016). For example, only one or two of many 3D conformations may translate to bioactivity, and there is no guarantee that the conformation picked for model building is the active conformation (Sukumar et al. 2011).

In addition, class separability, as used in this study, refers to the distinctness between class labels (active and inactive) in the t-SNE visualization (Fig. 2). The visualization offers vital insights into the prospective performance of our models. The plots for 2D descriptors and ECFP fingerprints displayed fairly distinct clusters, signifying good separation between active and inactive compounds. This separation suggests the features effectively capture the crucial differences between the class labels. Conversely, the less distinct distribution in the t-SNE plot of the 3D descriptors implies they might not distinguish active from inactive molecules as effectively, potentially explaining the lower performance of the models trained on the 3D descriptors.

Indeed, studies have shown in many cases that models trained on molecular representations such as 2D descriptors and ECFP fingerprint outperform those trained on representations that have 3D descriptors (Ahmed et al. 2021; Asahara & Miyao 2022; Bahia et al. 2023; Nettles et al. 2006; Orosz et al. 2022; Roy & Roy 2009, 2008).

### Learning curve

**Fig. 6:**
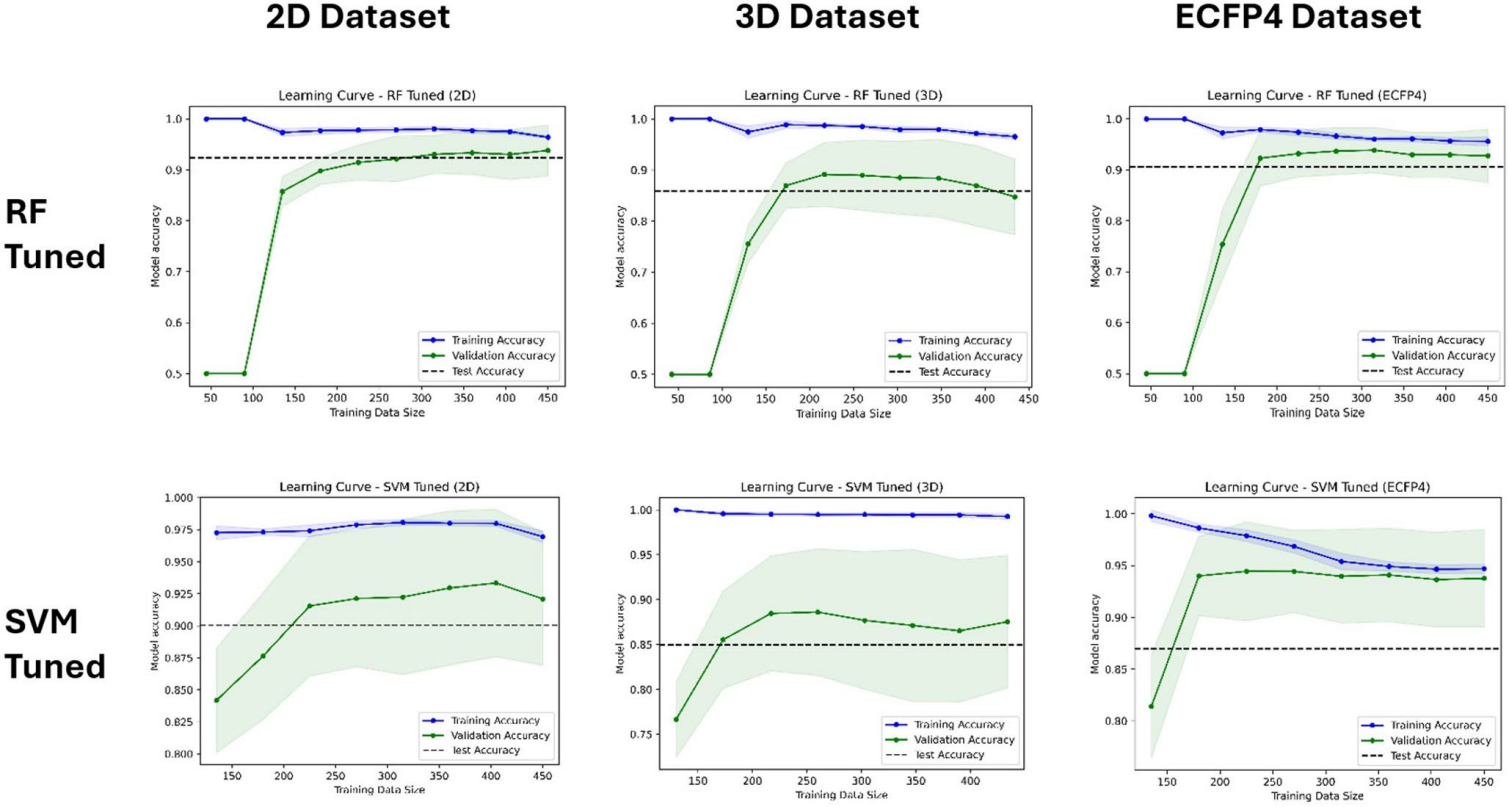
Learning curves for the tuned RF and tuned SVM models

To assess models’ behaviour, the learning curve tool from Scikit-learn was used to generate data to plot models’ learning curves, with balanced accuracy as the scoring choice (Fig. 6). Learning curves are vital tools for assessing overfitting in machine learning models (Jabbar & Khan 2014). These curves depict how a model's performance evolves as the amount of training data increases. They offer insights into whether the model is overfitting or underfitting. Regardless of the training scores' values during cross-validation, the true gauge of a model's performance lies in evaluating its predictive power on validation set and unseen test set. High training scores coupled with low validation or test scores often indicate overfitting, where the model memorizes the training data but fails to generalize well to unseen data which is a manifestation of the low bias-high variance problem. Conversely, low training and validation scores typically signify underfitting, implying the model's failure to capture the underlying patterns in the data which is a reflection of the high bias-low variance problem. In this study, the learning curve for tuned RF models trained on 2D and ECFP4 datasets showed reduced overfitting tendencies compared to the 3D dataset (Fig. 6). Moreover, the training and validation metric reached a steady state where additional training data does not improve the model’s performance. This is suggestive that the training data size used for our tuned RF models is sufficient for model training. The RF models left untuned generally showed perfect accuracy regardless of training data size (Figure S3), which may be suggestive of overfitting where the model fully memorized the training data. It can be said that tuning the models was beneficial for reducing overfitting tendencies. The learning curves for the SVM models showed more desirable trends in model trained on ECFP4 dataset compared to the 2D and 3D datasets. While some tuned SVM models showed good learning curves with reduced overfitting tendencies, they appear not to match up to the tuned RF models.

### Y-randomization

To ensure that the developed models do not arise from chance, y-randomization is performed through scrambled biological activity of the three datasets. The scrambled training datasets are then used to train models as in the unscrambled, performing tenfold cross validation and testing the model with unseen test set. The models performed poorly on the various metric evaluation as seen in Table 8 (balanced accuracy, F1 score, MCC and AUC). This y-randomization result validates that the model performance seen in the unscrambled data did not arise by chance.

**Table 8:** Model performance on test set upon Y-randomization

		Accuracy	Balanced accuracy	F1	MCC	AUC
2D	RF	0.71	0.55	0.28	0.11	0.48
	RF Tuned	0.71	0.56	0.30	0.12	0.51
	SVM	0.68	0.54	0.29	0.08	0.47
	SVM Tuned	0.67	0.51	0.23	0.02	0.46
3D	RF	0.66	0.51	0.23	0.01	0.53
	RF Tuned	0.70	0.54	0.27	0.08	0.55
	SVM	0.74	0.55	0.27	0.12	0.61
	SVM Tuned	0.70	0.53	0.25	0.07	0.59
ECFP4	RF	0.68	0.52	0.23	0.03	0.55
	RF Tuned	0.68	0.52	0.23	0.03	0.57
	SVM	0.68	0.52	0.23	0.03	0.57
	SVM Tuned	0.70	0.53	0.24	0.06	0.53

### Applicability domain and virtual screening

The model trained on ECFP4 was used to screen the Drugbank data (version 5.1.10) accessed via Probes and Drugs platform, to address drug repurposing. We retrieved the DrugBank dataset and have 10,455 drugs after ligand preparation. This set includes approved, investigational, experimental, and withdrawn drugs. To ensure reliable predictions, we defined the applicability domain for the RF tuned ECFP4 model. Defining the applicability domain is crucial for reliable predictions. It essentially carves out the safe zone for the model, identifying the chemical space where its predictions are trustworthy based on the training data (Gadaleta et al. 2016). This helps avoid using the model on structures too different from what it learned from, preventing misleading or inaccurate results. Using Euclidean distance, the applicability domain establishes a threshold of 8.25, representing the maximum distance a candidate drug can be from the training data for the model's predictions to be trustworthy. Consequently, we filtered the virtual screening data, removing any drug candidates exceeding this distance threshold. This filtration leaves us with 9625 drugs to subject to model prediction, suggesting a broad applicability across structurally diverse ligands.

**Fig. 7:**
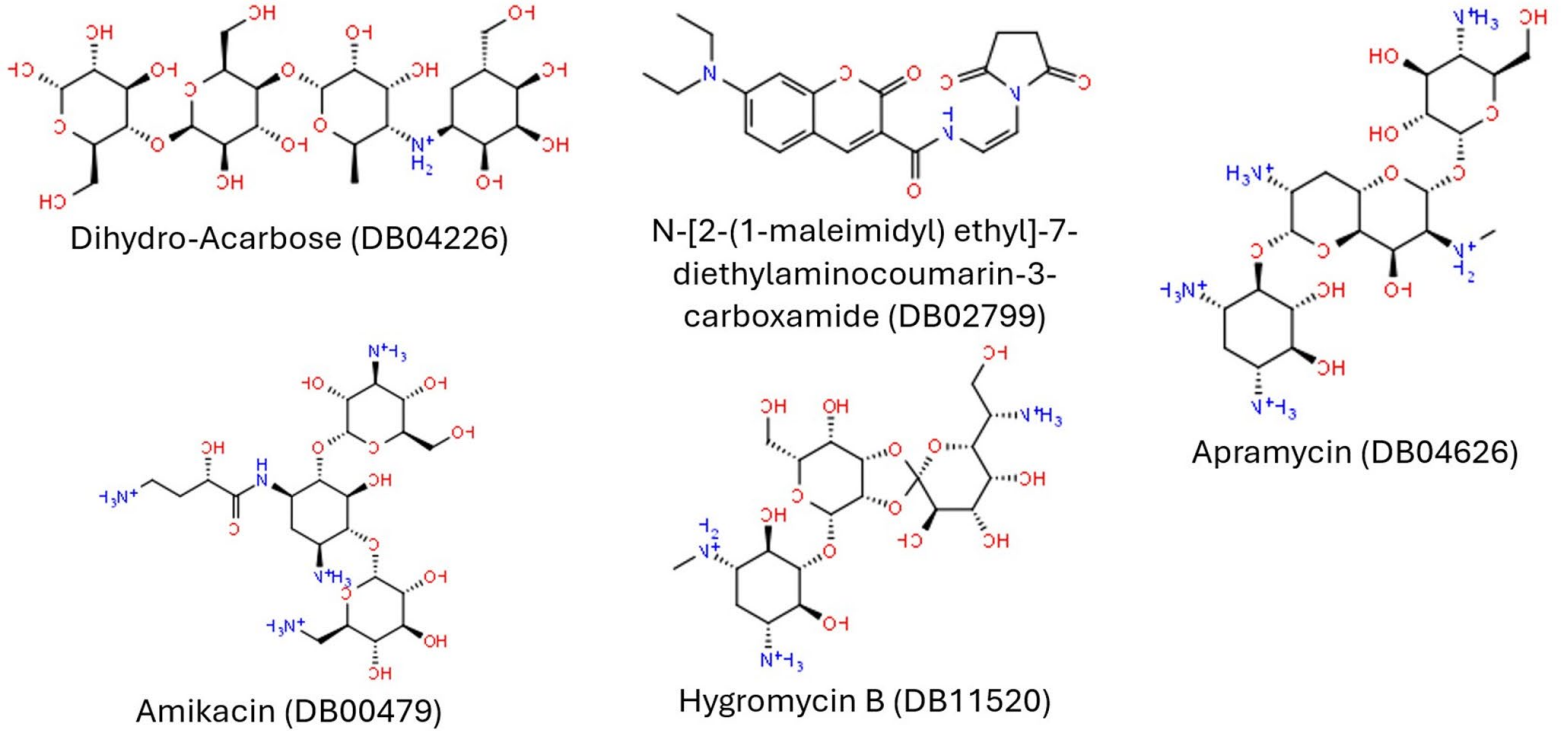
Identified hits from screening DrugBank database

Following the virtual screening process with the ECFP4 model, five hits not captured in the training data were identified. They are Dihydro-Acarbose (DB04226), N-[2-(1-maleimidyl) ethyl]-7-diethylaminocoumarin-3-carboxamide (DB02799), Hygromycin B (DB11520), Apramycin (DB04626) and Amikacin (DB00479). The structures of these compounds are presented in Fig. 7.

### Molecular docking and molecular dynamics simulation

An in-silico validation approach was employed to evaluate the interaction between the identified hits and a structural model of intestinal α-glucosidase (PDB ID: 2QMJ) which is a representative of intestinal α-glucosidases involved in dietary carbohydrate digestion. This structure was selected for its relevance to type 2 diabetes management and its availability in complex with the known inhibitor acarbose. Molecular docking and molecular dynamics simulations were conducted on each protein–ligand complex, revealing various non-covalent interactions including hydrogen bonding, water bridges, salt bridges, pi-cations, and hydrophobic interactions. The 2D representation of protein–ligand interactions, depicted in Figure S4, elucidates the intricate patterns of interactions. Docking scores ranging from -13.62 to − 2.86 kcal/mol suggest favourable interactions for most compounds with acarbose showing the most favourable score of − 13.62 kcal/mol and N-[2-(1-maleimidyl) ethyl]-7-diethylaminocoumarin-3-carboxamide exhibiting the least favourable higher score of − 2.86 kcal/mol (Table S1). While acknowledging the limitations of docking scores as a sole metric for evaluating interactions, further assessment using MM-GBSA revealed free binding energies ranging from − 15.03 to − 55.28 kcal/mol (Table S1), affirming the favourable nature of these interactions in an implicit solvent environment. However, to judge the stability of these interactions in an explicit solvent environment, molecular dynamics simulations were performed over a 150 ns period.

**Fig. 8:**
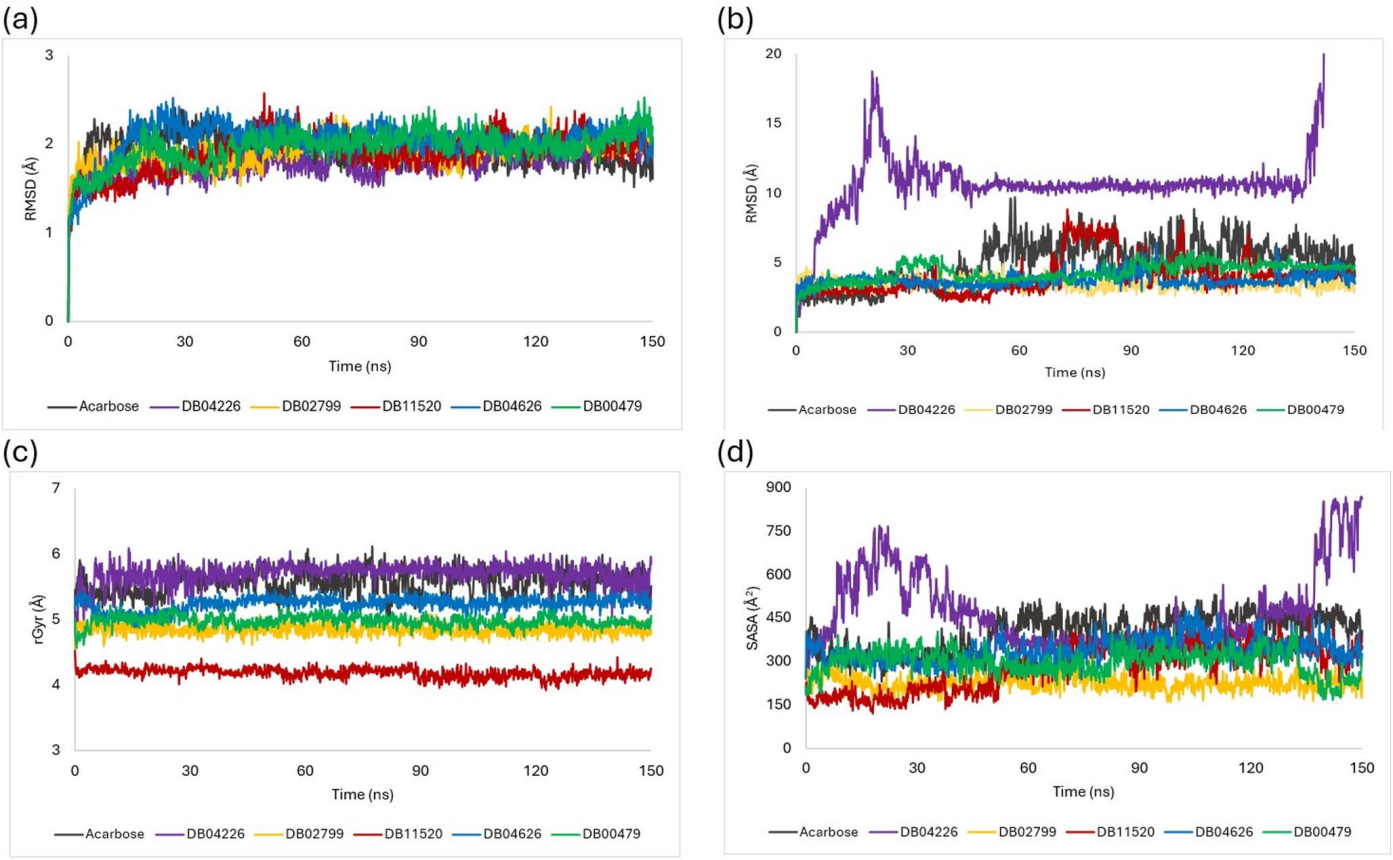
Root Mean Squared Deviation (RMSD) of **a** proteins in the protein–ligand complexes and **b** ligands with respect to proteins in the protein–ligand complexes. **c** The radius of gyration (rGyr) of ligands within the complex **d** Solvent accessible surface area (SASA)

The resulting analysis of the simulations indicates the overall stability of the protein–ligand complexes, with an average protein RMSD of about 1.93 Å throughout the simulation duration (Fig. 8a). Ligand RMSD measurements revealed that the ligands (DB02799, DB11520, DB04626, DB00479 and Acarbose) maintained close proximity to the protein, with an average RMSD of about 3.90 Å, although dihydro-acarbose (DB04226) exhibited lesser stability with higher RMSD (Fig. 8b). Observation of the trajectory timeline animation of DB04226 revealed it remained around the outer edge of the binding site, ultimately dislodging from the binding site after about 140 ns.

Figure 8c presents the radius of gyration (rGyr) of the ligands across the simulation time. We observed that rGyr values are in decreasing order: DB04226 > Acarbose > DB04626 > DB00479 > DB02799 > DB11520. The rGyr may give insight into the flexibility and conformational changes of ligands upon binding. A low rGyr might suggest the ligand adopts a more compact and potentially rigid conformation upon binding, potentially contributing to a stable complex (Mir & Nayak 2022).

The solvent-accessible surface area (SASA) is used to evaluate the accessibility of the ligand's surface to the solvent molecules throughout the simulation. Reduced and stable SASA trend indicates more extensive interactions between the protein and ligand, potentially leading to higher binding affinity (Mahaboob Ali et al. 2024). As observed in our study, the hits maintained a minimal and steady SASA trend till the end of the simulation, suggesting stable binding with α-glucosidase. However, DB04226 showed an increased SASA at the start of the simulation until it stabilized at about 60 ns, remaining so until it increased again close to the end. This suggests a short-lived stability in DB04226's interaction with the target compared to the other ligands.

Examination of ligand–protein contacts over simulation trajectories illustrated varying degrees of interaction, with amikacin (DB00479) and apramycin (DB04626) showing widespread non-covalent contacts occurring over 70% of simulation period, whereas hygromycin B (DB11520) displayed contacts with lower percentage of occurrence. Notably, DB02799 and DB04226 exhibited localized contacts, with DB02799 demonstrating enhanced stability compared to DB04226, as depicted in Figure S5. Collectively, these findings from molecular docking and dynamics simulations underscore the potential of the identified compounds as inhibitors of intestinal α-glucosidase, although further validation through in-vitro experiments are warranted for comprehensive assessment.

**Fig. 9:**
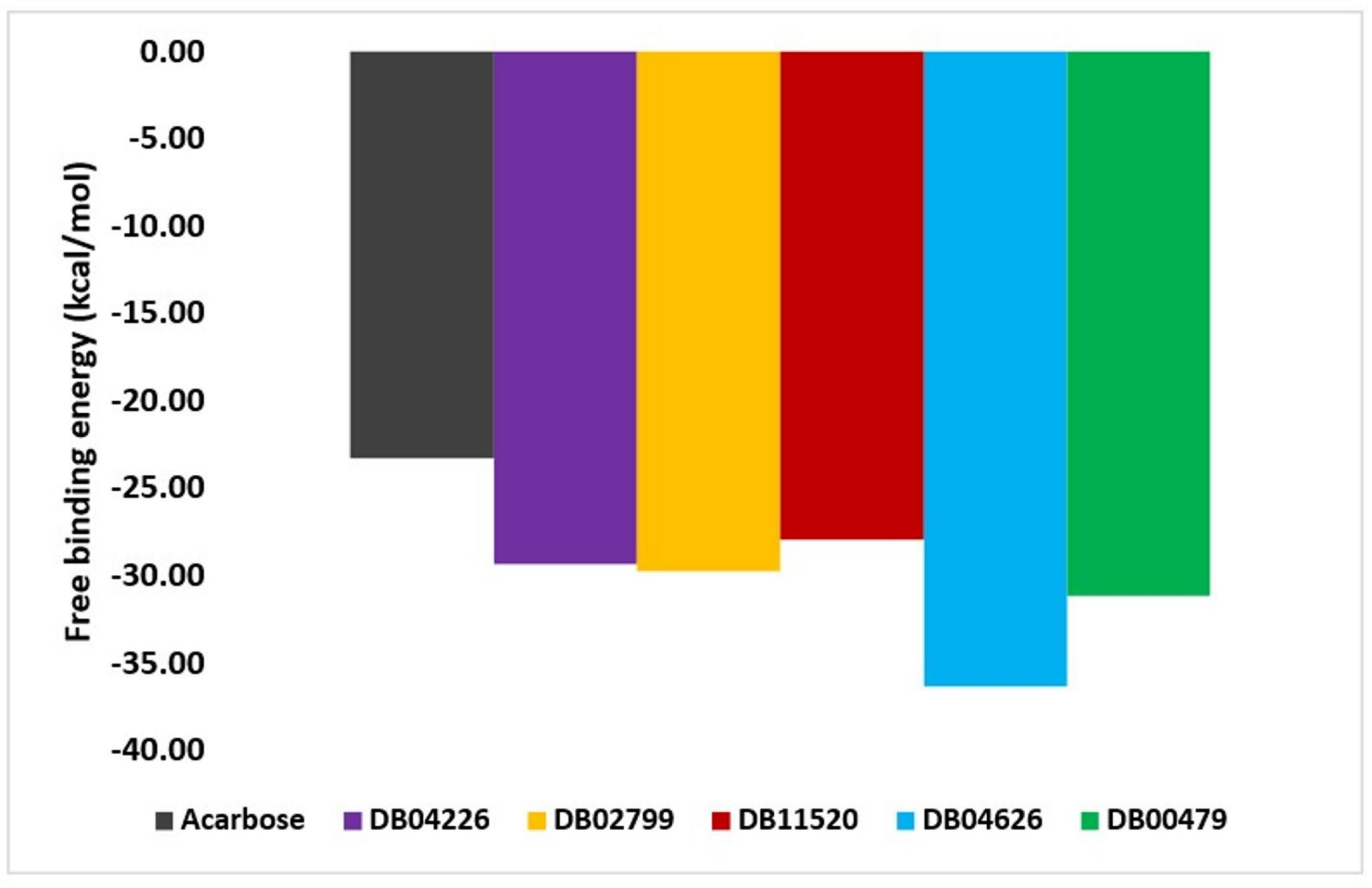
Post-MDS MM-GBSA calculations showing the average binding free energies of the protein–ligand complexes across simulation trajectories

The MM-GBSA analysis of the post-MD simulation trajectories provided estimates of the binding free energies for the hits and the reference compound, acarbose (Fig. 9). Among the screened candidates, DB04626 exhibited the most favourable binding energy average (− 36.34 kcal/mol), followed closely by DB02799 (− 29.75 kcal/mol) and DB04226 (− 29.33 kcal/mol). These values indicate stronger predicted binding affinities than Acarbose (− 23.29 kcal/mol), a known α-glucosidase inhibitor. This suggests that these compounds, particularly DB04626, may possess comparable or superior inhibitory potential and warrant further investigation as repurposing candidates.

## Conclusion

This study highlights the potential of machine learning techniques to develop predictive models for identifying α-glucosidase inhibitors, which are essential for managing diabetes. Models trained on 2D descriptors and ECFP4 fingerprints outperformed those using 3D descriptors. Furthermore, we demonstrate the potential utility of the model trained on ECFP4 fingerprints for drug repurposing by screening the Drugbank database, identifying potential hit compounds for α-glucosidase inhibition. Molecular dynamics simulations further validate the potential of these compounds as inhibitors, providing insights into the stability of their interactions with α-glucosidase. Our work suggests a direction for using ML-driven QSAR modelling applied to a structurally diverse ligand set, screening for potential drug repurposing candidates. This approach could be a cost-effective path for early-stage discovery of potential antidiabetic agents. However, this study is limited by its reliance on in-silico validation alone. The predictive performance and biological relevance of the identified compounds need to be confirmed through in vitro and in vivo experiments.

## Electronic supplementary material

Below is the link to the electronic supplementary material.

Supplementary Material 1

Supplementary Material 2

## Data Availability

Data is provided within the manuscript or supplementary information files Python codes for model training are available at https://github.com/shinoxide/ML_QSAR_model.
