# Supplementary material for: Machine learning prediction of intestinal α-glucosidase inhibitors using a diverse set of ligands: a drug repurposing effort with drugBank database screening
Source: In Silico Pharmacol. 2025 Jun 25;13(2):95. doi: 10.1007/s40203-025-00384-8 (PMC12198089; doi:10.1007/s40203-025-00384-8)
Supplement: Supplementary file 2 — Supplementary Material 2 [file 40203_2025_384_MOESM2_ESM.docx]

**SUPPLEMENTARY INFORMATION**

Machine Learning Prediction of Intestinal α-Glucosidase Inhibitors Using a Diverse Set of Ligands: A Drug Repurposing Effort with DrugBank Database Screening

Adeshina I. Odugbemi^1,4^, Clement Nyirenda^2^, Alan Christoffels^1^, Samuel A. Egieyeh^3,4^*

^1^ South African Medical Research Council Bioinformatics Unit, South African National Bioinformatics Institute, University of the Western Cape, Bellville, Cape Town 7535, South Africa.

^2^ Department of Computer Science, University of the Western Cape, Cape Town 7535, South Africa.

^3^ School of Pharmacy, University of the Western Cape, Bellville, Cape Town 7535, South Africa.

^4^ National Institute for Theoretical and Computational Sciences (NITheCS), South Africa.

* Corresponding author

E-mail: [segieyeh@uwc.ac.za](mailto:segieyeh@uwc.ac.za)

Contents

[Figure S1: Confusion Matrices for all test sets across the 2D and 3D descriptors, and the ECFP dataset 3](#_Toc199527473)

[Figure S2: Confusion Matrices for all training sets across the 2D and 3D descriptors, and the ECFP dataset 4](#_Toc199527474)

[Figure S3: Learning curves for the untuned RF and SVM models 5](#_Toc199527475)

[Table S1: Docking scores and MM-GBSA free binding energies of virtual screening hits. 5](#_Toc199527476)

[Figure S4: The 2D ligand interaction diagram of α-glucosidase (2QMJ) binding with (a) Dihydro-Acarbose (DB04226) (b) N-[2-(1-maleimidyl) ethyl]-7-diethylaminocoumarin-3-carboxamide (DB02799) (c) Hygromycin B (DB11520) (d) Apramycin (DB04626) (e) Amikacin (DB00479) 7](#_Toc199527477)

[Figure S5: Ligand-protein percentage contacts for (a) Dihydro-Acarbose (DB04226) (b) N-[2-(1-maleimidyl) ethyl]-7-diethylaminocoumarin-3-carboxamide (DB02799) (c) Hygromycin B (DB11520) (d) Apramycin (DB04626) (e) Amikacin (DB00479) and (f) Acarbose for at least 30% of the simulation time. 8](#_Toc199527478)

|  | 2D Dataset | 3D Dataset | ECFP4 Dataset |
| --- | --- | --- | --- |
| RF | 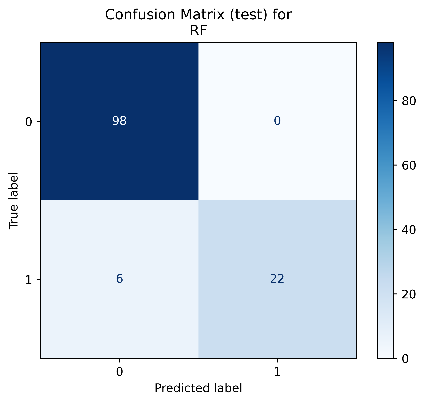 | 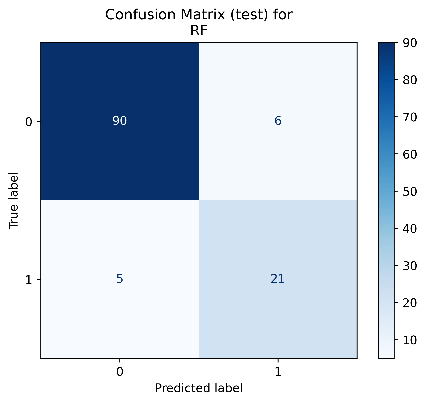 | 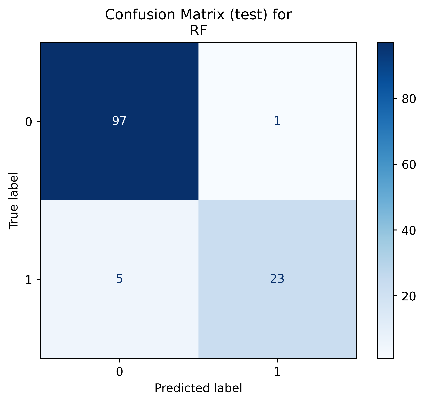 |
| RF Tuned | 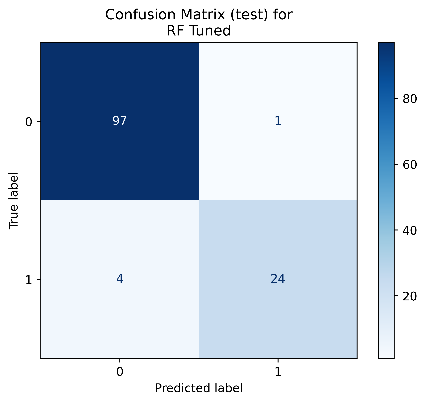 | 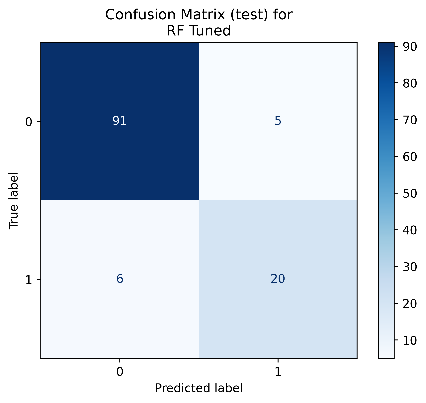 | 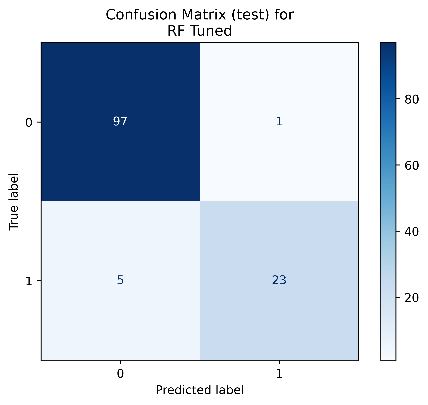 |
| SVM | 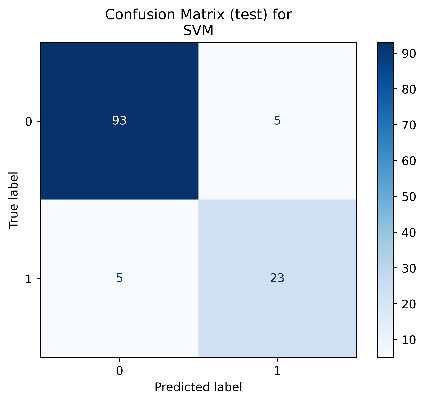 | 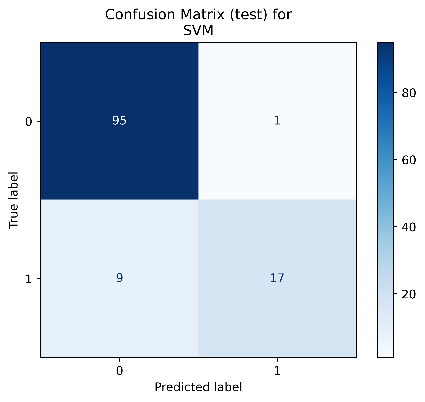 | 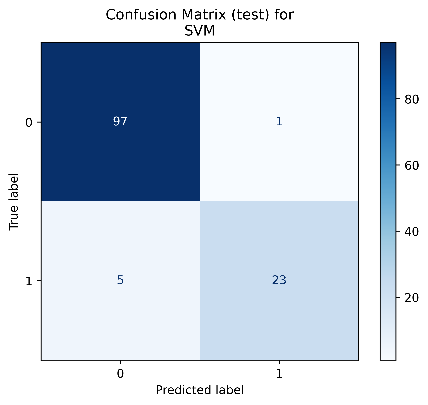 |
| SVM Tuned | 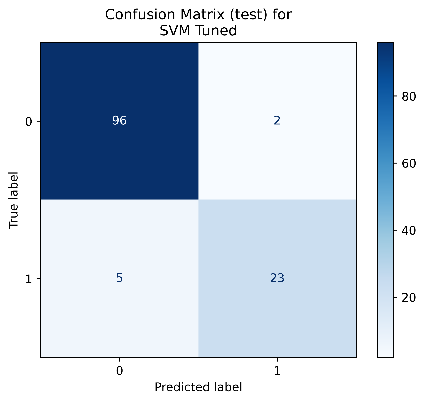 | 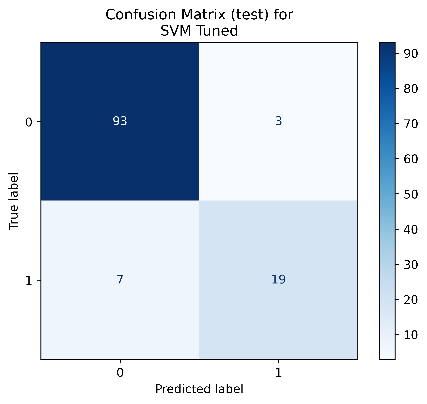 | 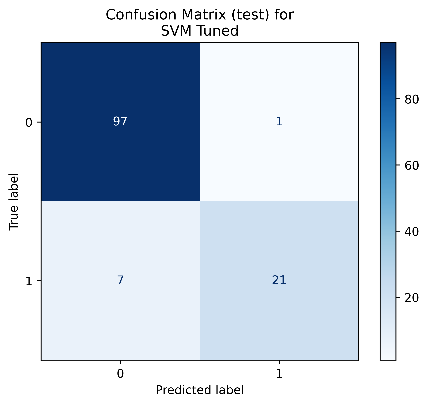 |

# Figure S1: Confusion Matrices for all test sets across the 2D and 3D descriptors, and the ECFP dataset

|  | 2D Dataset | 3D Dataset | ECFP4 Dataset |
| --- | --- | --- | --- |
| RF | 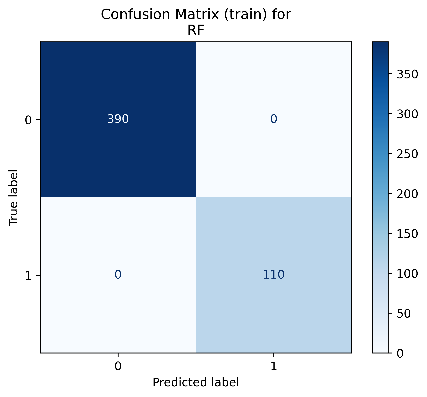 | 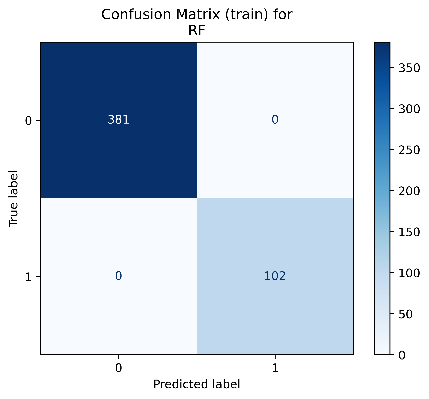 | 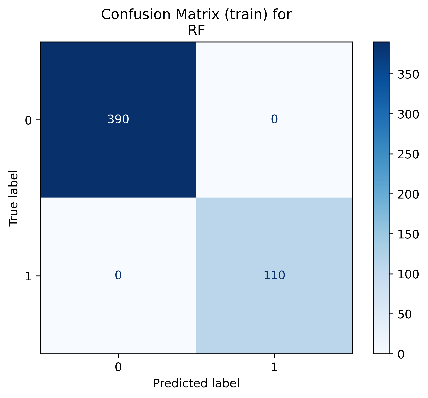 |
| RF Tuned | 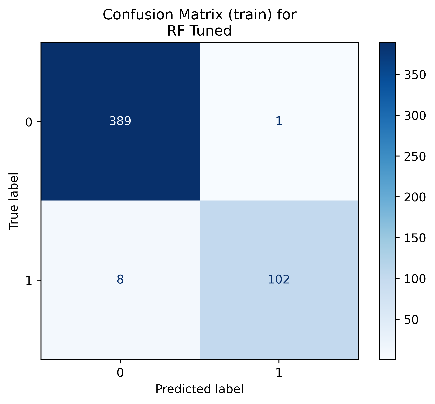 | 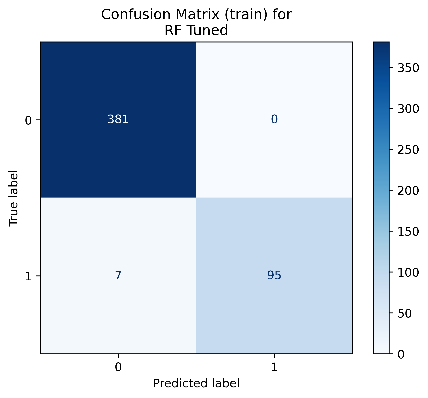 | 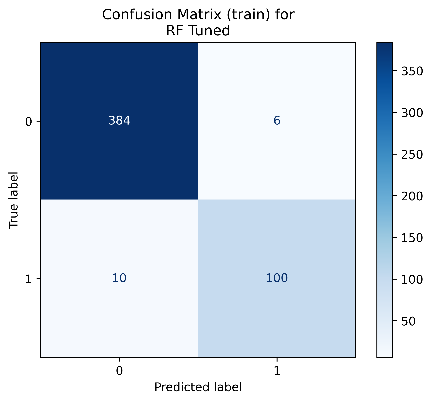 |
| SVM | 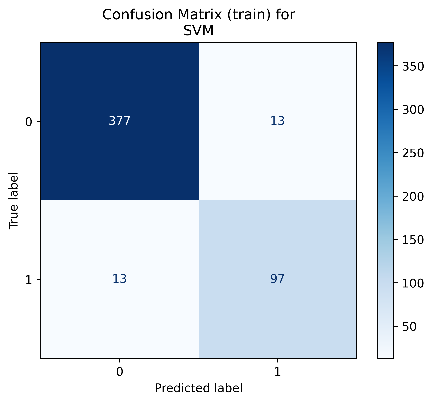 | 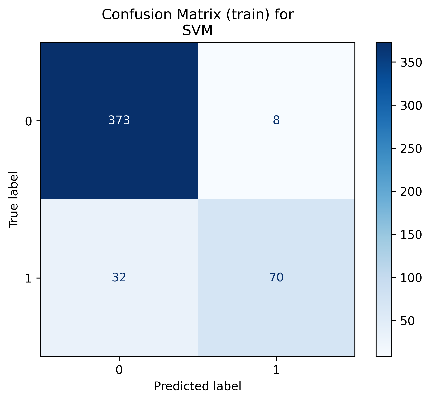 | 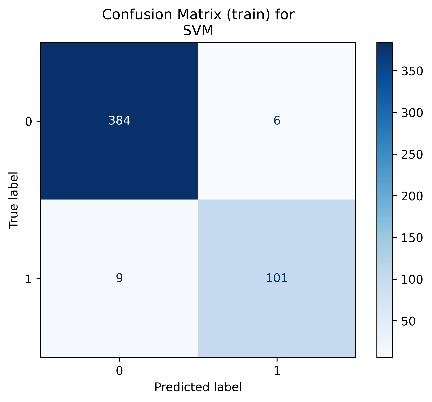 |
| SVM Tuned | 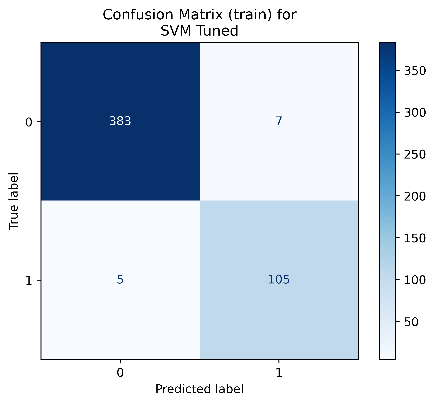 | 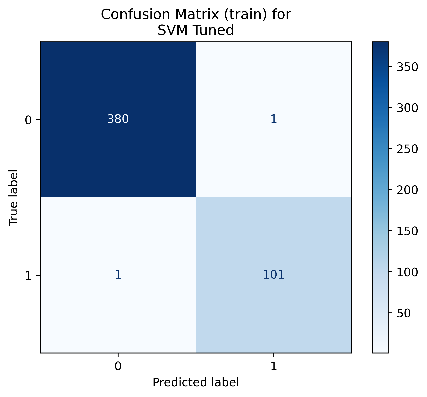 | 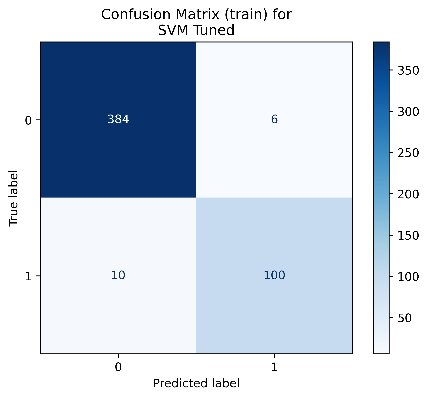 |

# Figure S2: Confusion Matrices for all training sets across the 2D and 3D descriptors, and the ECFP dataset

|  | 2D Dataset | 3D Dataset | ECFP4 Dataset |
| --- | --- | --- | --- |
| RF | 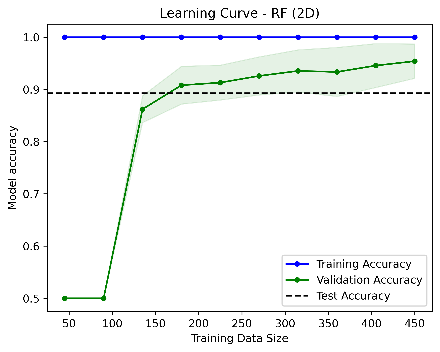 | 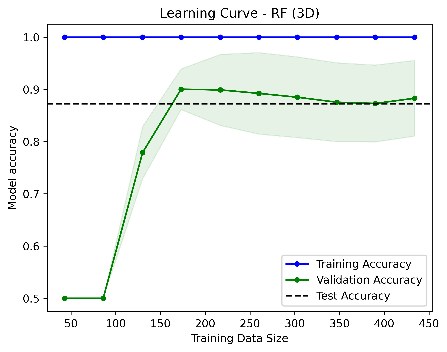 | 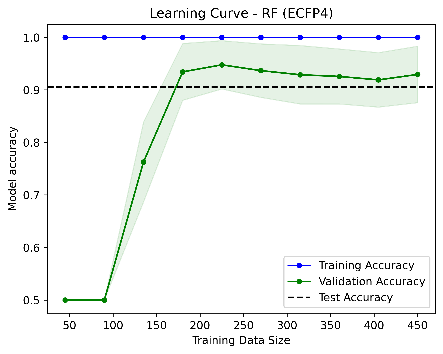 |
| SVM | 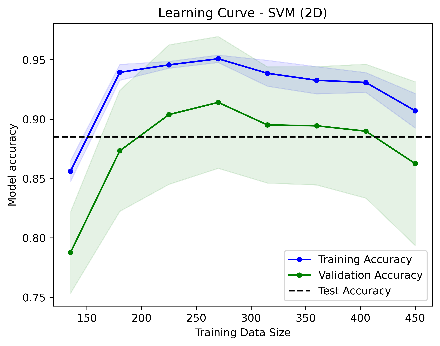 | 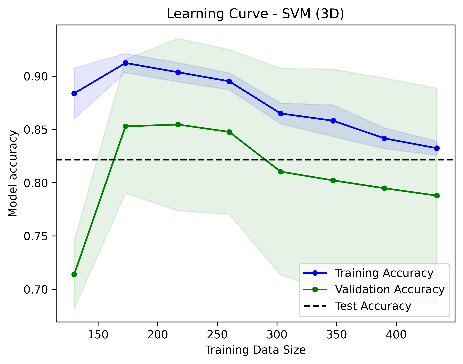 | 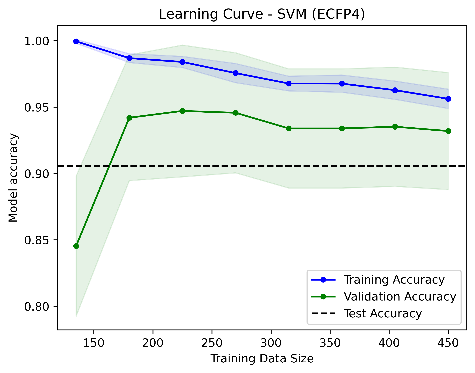 |

# Figure S3: Learning curves for the untuned RF and SVM models

# Table S1: Docking scores and MM-GBSA free binding energies of virtual screening hits.

| Drug name | DrugBank ID | Docking Score (Kcal/mol) | Post-Docking MMGBSA dGbind (Kcal/mol) | Post-MDS MMGBSA dGbind (Kcal/mol) |
| --- | --- | --- | --- | --- |
| Dihydro-Acarbose | DB04226 | -10.94 | -27.03 | -29.33±2.43 |
| N-[2-(1-maleimidyl)ethyl]-7-diethylaminocoumarin-3-carboxamide | DB02799 | -2.86 | -15.03 | -29.75±1.66 |
| Hygromycin B | DB11520 | -13.28 | -22.93 | -27.95±2.11 |
| Apramycin | DB04626 | -9.77 | -55.28 | -36.34±2.24 |
| Amikacin | DB00479 | -13.32 | -39.43 | -31.15±2.24 |
| Acarbose |  | -13.60 | -41.74 | -23.29±2.39 |

| (a) | 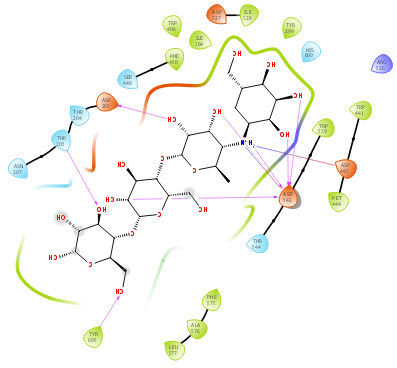 | 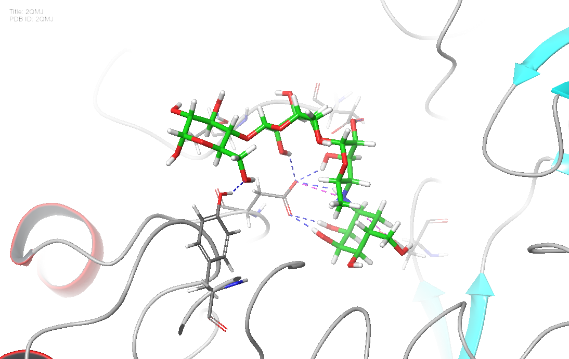 |
| --- | --- | --- |
| (b) | 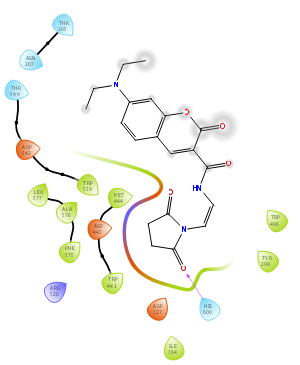 | 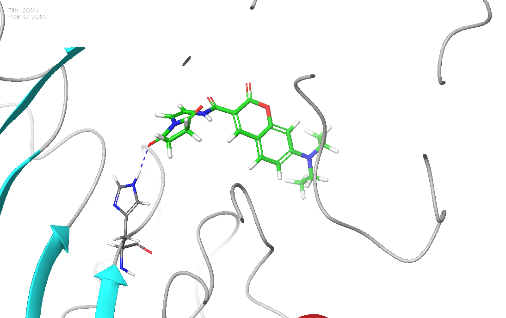 |
| (c) | 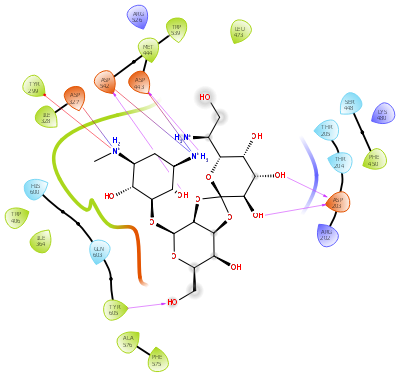 | 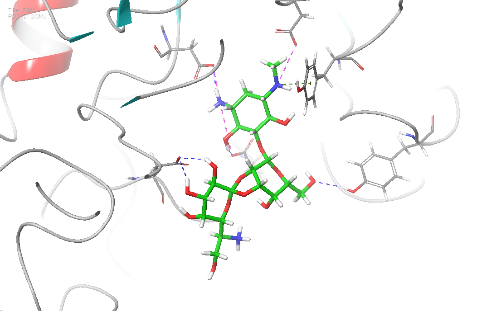 |
| (d) | 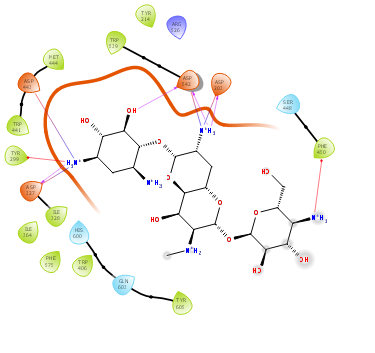 | 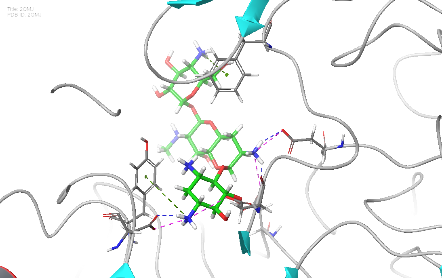 |
| (e) | 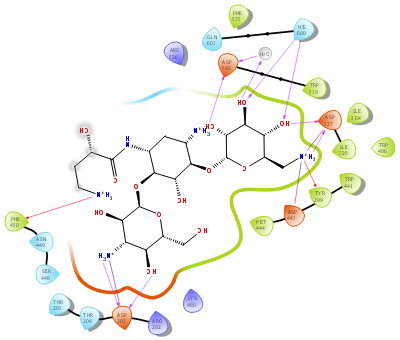 | 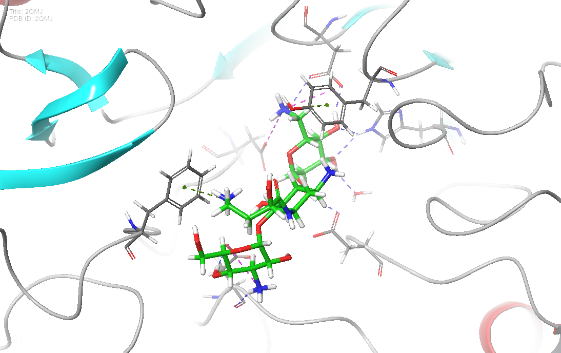 |
| (f) | 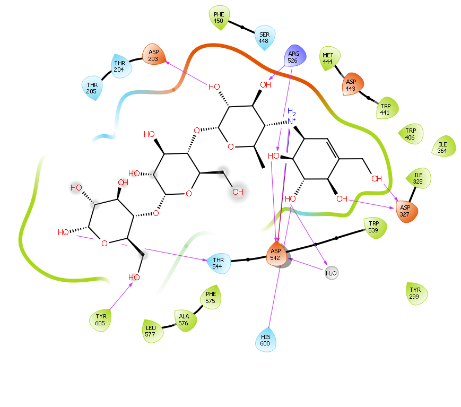 | 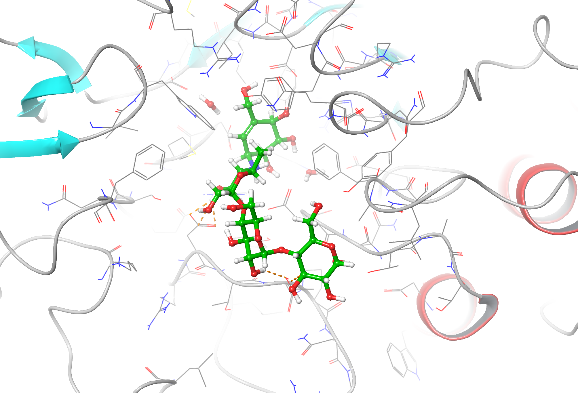 |
|  | 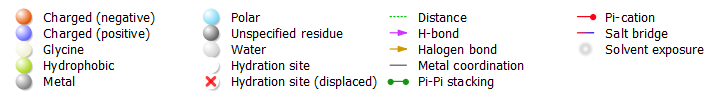 | |

# Figure S4: The 2D ligand interaction diagram of α-glucosidase (2QMJ) binding with (a) Dihydro-Acarbose (DB04226) (b) N-[2-(1-maleimidyl) ethyl]-7-diethylaminocoumarin-3-carboxamide (DB02799) (c) Hygromycin B (DB11520) (d) Apramycin (DB04626) (e) Amikacin (DB00479) (f) Acarbose

| (a)  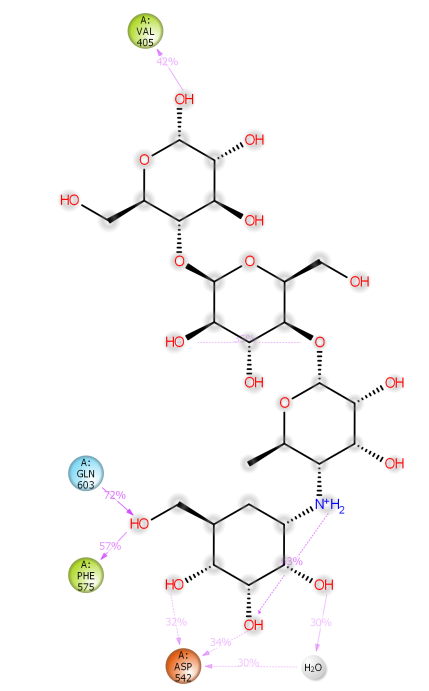 | (b)  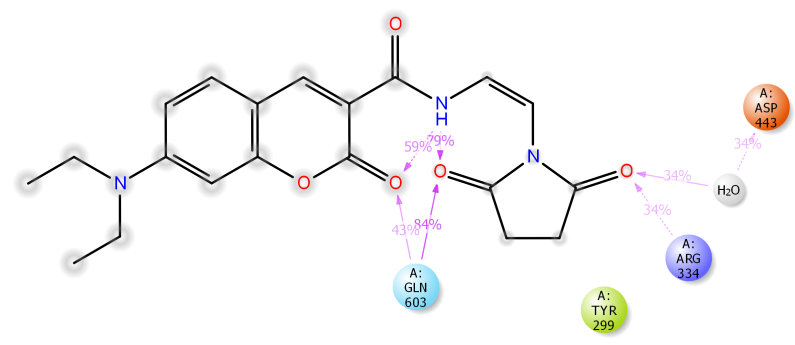 |
| --- | --- |
|  | (c)  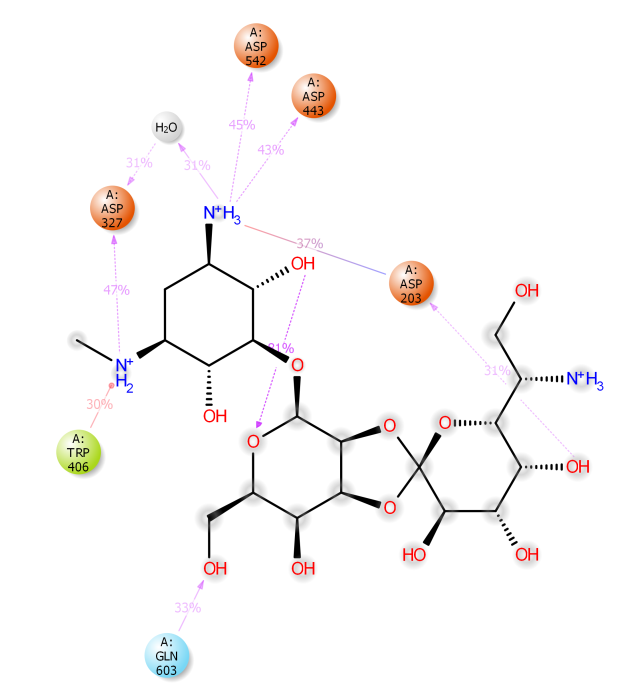 |
| (d)  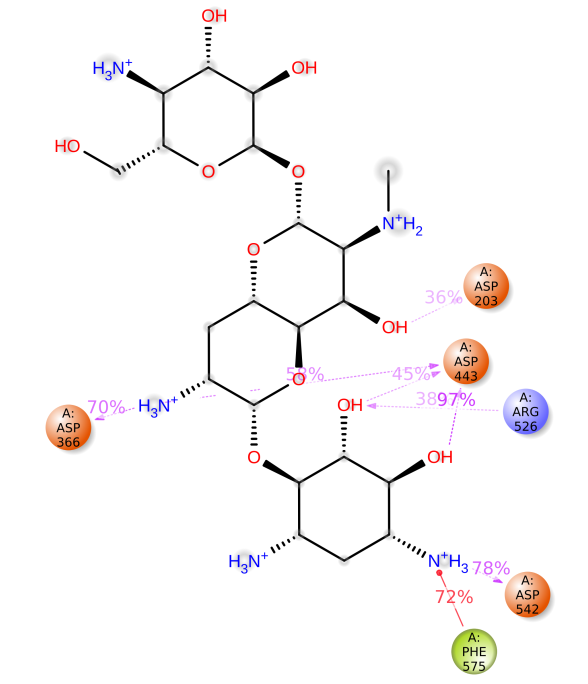 | (e)  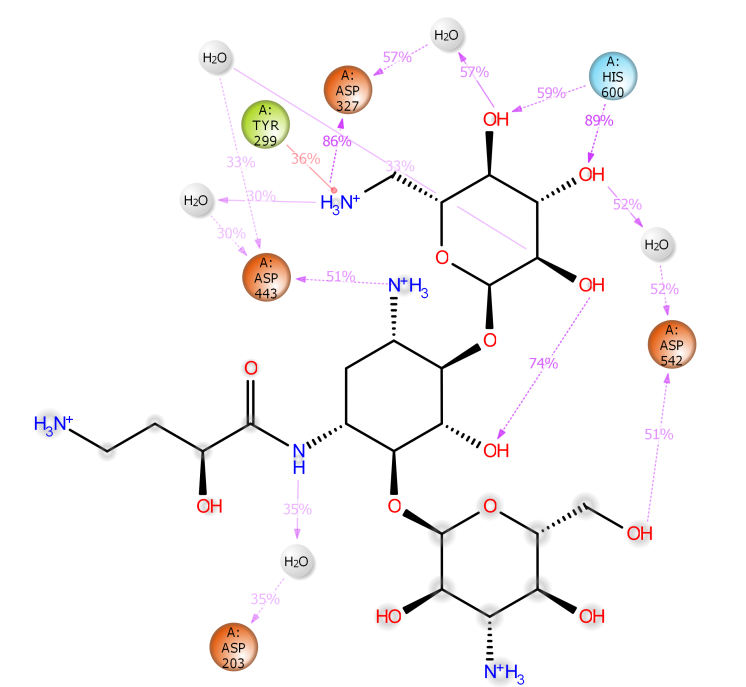 |
| (f)  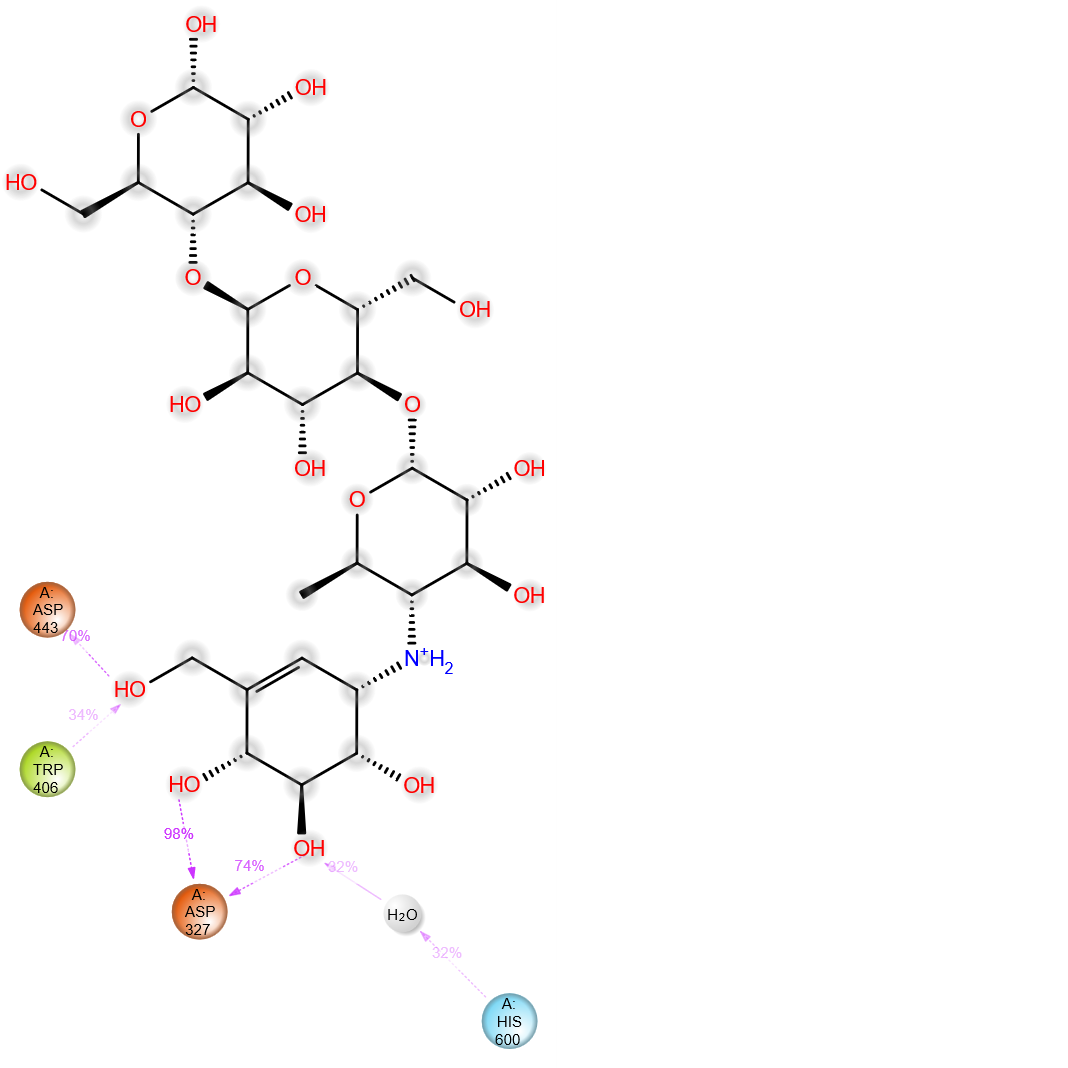 | 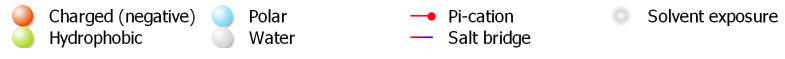 |

# Figure S5: Ligand-protein percentage contacts for (a) Dihydro-Acarbose (DB04226) (b) N-[2-(1-maleimidyl) ethyl]-7-diethylaminocoumarin-3-carboxamide (DB02799) (c) Hygromycin B (DB11520) (d) Apramycin (DB04626) (e) Amikacin (DB00479) and (f) Acarbose for at least 30% of the simulation time.
